# SUR1-TRPM4 is expressed in human epilepsy and promotes neuron hyperactivity and seizures in rodents

**DOI:** 10.1093/brain/awaf435

**Published:** 2025-11-15

**Authors:** Mitchell B Moyer, Svetlana Ivanova, Kaspar Keledjian, Matthew Kreinbrink, Jenna Langbein, Penghua Yang, Darrian McAfee, Ujwal Boddeti, Ziam Khan, Jemima Olu-Owotade, Timothy Zhang, David R Benavides, Joshua Diamond, Kareem Zaghloul, Muznabanu Bachani, Volodymyr Gerzanich, J Marc Simard, Alexander Ksendzovsky

**Affiliations:** Department of Neurosurgery, University of Maryland School of Medicine, Baltimore, MD 21201, USA; Department of Neurosurgery, University of Maryland School of Medicine, Baltimore, MD 21201, USA; Department of Neurosurgery, University of Maryland School of Medicine, Baltimore, MD 21201, USA; Department of Neurosurgery, University of Maryland School of Medicine, Baltimore, MD 21201, USA; Department of Neurosurgery, University of Maryland School of Medicine, Baltimore, MD 21201, USA; Department of Neurosurgery, University of Maryland School of Medicine, Baltimore, MD 21201, USA; Department of Neurosurgery, University of Maryland School of Medicine, Baltimore, MD 21201, USA; Department of Neurosurgery, University of Maryland School of Medicine, Baltimore, MD 21201, USA; Department of Neurosurgery, University of Maryland School of Medicine, Baltimore, MD 21201, USA; Department of Neurosurgery, University of Maryland School of Medicine, Baltimore, MD 21201, USA; Department of Neurology, University of Maryland School of Medicine, Baltimore, MD 21201, USA; Department of Neurology, University of Maryland School of Medicine, Baltimore, MD 21201, USA; Surgical Neurology Branch, National Institute of Neurologic Disorders and Stroke, National Institutes of Health, Bethesda, MD 20892, USA; Surgical Neurology Branch, National Institute of Neurologic Disorders and Stroke, National Institutes of Health, Bethesda, MD 20892, USA; Department of Neurosurgery, University of Maryland School of Medicine, Baltimore, MD 21201, USA; Department of Neurosurgery, University of Maryland School of Medicine, Baltimore, MD 21201, USA; Department of Neurosurgery, University of Maryland School of Medicine, Baltimore, MD 21201, USA; Department of Pathology, University of Maryland School of Medicine, Baltimore, MD 21201, USA; Department of Physiology, University of Maryland School of Medicine, Baltimore, MD 21201, USA; Department of Neurosurgery, University of Maryland School of Medicine, Baltimore, MD 21201, USA

**Keywords:** SUR1-TRPM4, epilepsy, seizure threshold, ion channels, channelopathy, hyperexcitation

## Abstract

One-third of epilepsy patients do not achieve sufficient seizure freedom with current standard anti-seizure medications. Better understanding of the pathological mechanisms contributing to epileptogenesis is thus necessary to improve current therapies. SUR1-TRPM4 is a depolarizing ion channel minimally expressed in a healthy brain that is upregulated *de novo* in neurons and glia after epileptogenic CNS injuries such as traumatic brain injury and stroke. However, its role in epilepsy is not well understood.

Here, we demonstrate using immunofluorescent microscopy that SUR1-TRPM4 expression is elevated in neurons within an electrographically sorted human epileptic brain compared with a non-epileptic brain obtained after resection from six drug-resistant temporal lobe epilepsy patients. Additionally, we utilized immunofluorescence and co-immunoprecipitation to observe that SUR1-TRPM4 is upregulated within the hippocampus and temporal cortex in mice after pentylenetetrazol (PTZ) kindling, a chronic model of rodent epilepsy. Pharmacologic inhibition of SUR1-TRPM4 using either the US Food and Drug Administration (FDA)-approved drug glyburide or 9-phenanthrol, as well as either constitutive or neuron-specific knockout of this channel, attenuated chronic seizure development in this model. Exogenous overexpression of SUR1-TRPM4 by plasmid transfection in neurons *in vitro* increased neuronal hyperexcitability in response to low Mg^2+^ stimulation, while pharmacologic inhibition of endogenous TRPM4 attenuated neuronal population hyperexcitation.

Collectively, our results reveal that elevated SUR1-TRPM4 expression found in human and rodent epileptic neurons promotes chronic seizures by increasing neuronal excitation. These findings directly support clinical investigation of SUR1-TRPM4 inhibitors as potential anti-seizure therapies in epilepsy patients and suggest further investigations into the contribution of SUR1-TRPM4 to seizures induced by specific epileptogenic insults, such as traumatic brain injury (TBI), are warranted.

## Teaser

Moyer *et al.* identified the SUR1-TRPM4 channel as a driver of neuronal hyperexcitability in epilepsy. They found this channel to be upregulated in human and rodent epileptic brain tissue, and showed that blocking or removing it in rodents attenuated chronic seizure development, suggesting its potential as a therapeutic target.

## Introduction

Epilepsy, one of the most prevalent neurological disorders in the USA, affects approximately 1.2% of the population (3.4 million people).^1^ Epilepsy is characterized by recurrence of spontaneous seizures driven by excessive brain excitability. Despite optimal medical treatment, nearly 30% of patients are resistant to anti-seizure medications (ASMs).^2^ Further, ASMs often lead to significant cognitive side effects and poor quality of life, which may worsen seizure control due to non-compliance.^2,3^ These side effects arise from ASMs targeting constitutively expressed proteins essential for normal neuronal and glial function.^3^ Therefore, identifying novel, epilepsy-specific therapeutic targets that are not constitutively expressed but emerge *de novo* during epileptogenesis is critical for advancing treatment strategies.

The SUR1-TRPM4 channel is an important therapeutic target in CNS disease.^4-10^ This hetero-octameric, non-selective, monovalent cation channel consists of four SUR1 subunits that bind to and regulate four TRPM4 subunits.^4^ TRPM4 subunits form a pore predominantly conducting Na^+^ under physiologic conditions, with binding of SUR1 to TRPM4 increasing channel open probability and Na^+^ influx in response to elevated intracellular Ca^2+^ or reduced ATP.^4,5^ The SUR1-TRPM4 channel is minimally expressed in the healthy brain but becomes pathologically upregulated *de novo* in neurons, glia and endothelial cells after injury.^4,9,10^ Upregulation has been observed in conditions linked to epileptogenesis, including stroke, traumatic brain injury, subarachnoid haemorrhage, intracerebral haemorrhage and neonatal hypoxia.^6,8-12^ Preclinical and clinical studies demonstrate the therapeutic benefits of SUR1-TRPM4 inhibition in these conditions.^4,7,8,13,14^ Astrocytic SUR1-TRPM4 contributes to cerebral oedema after injury through ion and water influx at astrocyte end-feet, while microglial expression supports the inflammatory response.^4,15,16^ However, the role of SUR1-TRPM4 in neurons remains unexplored.

Beyond its role in epilepsy-related pathologies, several mechanistic findings further suggest that SUR1-TRPM4 may be critical for epilepsy pathogenesis. First, TRPM4 regulates membrane potential in excitable cells, including neurons, by depolarization via inward cation flux.^4,5,17^ Second, SUR1 binding enhances TRPM4 activation in response to elevated Ca^2+^ and depleted ATP, as observed after brain injury.^4,5^ Third, basal interictal intracellular Ca^2+^ levels are elevated in chronic epilepsy models, and TRPM4 significantly contributes to Ca^2+^-mediated high frequency neuronal bursting in *ex vivo* seizure-like conditions.^18,19^ Based on these prior findings, we hypothesize that upregulated SUR1-TRPM4 in epileptic neurons amplifies depolarization and thus neuronal activity and enhances seizure susceptibility.^4,5^ Supporting this, rodent epilepsy models show neuronal SUR1-TRPM4 expression during the chronic phase after status epilepticus (SE), and SUR1-TRPM4 inhibition reduces spontaneous recurrent seizures post-SE.^20-22^ However, SUR1-TRPM4 expression in human epilepsy or non-SE rodent epilepsy models, and its role in epilepsies of diverse aetiologies, remain unclear.

In this study, we explored the novel hypothesis that increased neuronal SUR1-TRPM4 expression in epilepsy drives hyperexcitability and subsequently promotes chronic seizures. To test this, we examined SUR1-TRPM4 expression and function in human epilepsy and rodent epilepsy models independent of SE. We examined the effect of SUR1-TRPM4 overexpression and inhibition on seizure-like activity *in vitro* and evaluated the anti-seizure effect of SUR1-TRPM4 inhibition on pentylenetetrazol (PTZ) kindling using both pharmacologic and genetic approaches. Overall, we found SUR1-TRPM4 was upregulated in human epilepsy and non-SE animal epilepsy models where its expression promotes hyperexcitability and chronic seizures.

## Materials and methods

### Ethics statement

All patient samples were collected under an Institutional Review Board approved protocol (ClinicalTrials.gov identifier CT01273129) at the Clinical Center at the National Institutes of Health (NIH). Informed consent was obtained from all participants in the study. All animal use and collection of animal tissue samples was conducted under a protocol approved by the Institutional Animal Care and Use Committee (IACUC) at the University of Maryland. Animal experiments comply with the Animal Research: Reporting *In Vivo* Experiments (ARRIVE) guidelines 2.0 (https://arriveguidelines.org/arrive-guidelines) and other relevant guidelines and regulations as stipulated in the United States National Institutes of Health Guide for the Care and Use of Laboratory Animals.^23,24^

### Study design

The overall objectives of this study were to assess SUR1-TRPM4 channel expression in epilepsy and investigate its effects on neuronal hyperactivity and chronic seizure susceptibility. To that end, we utilized human tissues, *in vitro* and *in vivo* approaches to holistically characterize the role of the SUR1-TRPM4 channel in promoting chronic seizures and define SUR1-TRPM4 as a clinically important channel in epilepsy management. We used orthogonal approaches to support our key conclusions, including measuring SUR1-TRPM4 channel expression in both human and rodent epileptic brain tissue and using multiple pharmacologic and/or genetic methods of inhibition to measure the pro-seizure effects of SUR1-TRPM4 expression *in vitro* and *in vivo*. Investigators were not blinded during data acquisition or analysis, however, at least two investigators independently performed and corroborated Racine scoring, and data for microelectrode (MEA) and Ca^2+^ imaging experiments were acquired using automated methods (described below) and quantification methods were applied evenly across all groups. Additionally, Ca^2+^ imaging data were analysed using a custom MATLAB script to minimize potential for human bias. No randomization was performed for human tissue samples, as comparisons were made within patients between electrographically defined control and epileptic tissues. Animals and cell cultures were randomized into experimental groups, excluding protein knockout (KO) experiments where mice were assigned based on genotype. Mice of similar age, sex and genetic background were used across groups for *in vivo* experiments. Sample sizes were determined based on preliminary findings and previously published work.^25^ All experiments utilized at least three replicates, with the number of replicates for each experiment indicated in the text or figure legends. One treatment day was excluded for one treatment group for the *in vitro* microelectrode array (MEA) TRPM4 inhibition experiments, as no wells in that group met the 6 Hz mean firing rate pre-treatment baseline threshold predefined to exclude wells with low activity (treatment group identified in the figure legend). No other data-points were excluded from analysis, and there were no statistical outliers.

### Human patients/resection protocol

Tissue procurement and patient information for this study were as previously described.^25^ In brief, six patients [four males, age (mean ± standard deviation): 32.7 ± 8.45 years] with drug-resistant temporal lobe epilepsy with mesial temporal sclerosis underwent a surgical procedure for intracranial stereo EEG (iEEG) recordings in which platinum recording contacts (PMT Corporation) were implanted for seizure monitoring (Table 1). Continuous iEEG signals were sampled at 1000 Hz from all recording contacts using a Nihon Kohden EEG data acquisition system to identify seizure activity by an epileptologist. Based on epileptic electrographic patterns detected by iEEG, including epileptiform discharges during clinical seizures or interictally, we identified electrodes overlying the seizure onset zone (epileptogenic electrodes) and electrodes that were not involved in the seizure network (non-epileptic electrodes) but were within clinical bounds for resection. It is typical to remove areas of the brain that are not necessarily within the immediate seizure focus or network to gain access to the epileptogenic brain. Tissues within the clinical resection boundary underlying both epileptic and non-epileptic electrodes were resected, separated and compared. No tissue was removed solely for research purposes. The localization of the epileptic foci, in addition to electrographic detection, were corroborated using MRI, functional MRI (fMRI) and PET, and by seizure outcomes post-resection with every patient achieving near-seizure freedom (outcomes scored from Engel 1a-2a) (Table 1). For tissue fixation, we placed tissue samples into 4% paraformaldehyde (PFA) for 48 h directly from the operating room within 5 min of removal from the blood supply. We placed tissue samples into PBS solution and maintained them at 4°C until embedding in paraffin; 5 μm thick sections were prepared from paraffin embedded tissues.

**Table 1:** Patient information for human epilepsy brain samples used in this study

Patient	1	2	3	4	5	6
Sex	Male	Male	Female	Female	Male	Female
Age at seizure onset, years	3	23	20	13	17	37
Age at resection, years	39	33	28	30	21	45
Epilepsy duration, years	36	10	8	17	4	8
Epilepsy aetiology	Multi-factorial: febrile seizures and TBI	Periventricular nodular heterotopia (genetic)	Febrile seizures	TBI	Other- electrocution	Idiopathic
Seizure semiology	Dystonia followed by generalized tonic-clonic seizure	Repetitive vocalizations/automatisms	Complex partial seizure preceded by aura of oral tingling sensation	Pain in stomach followed by tonic seizure, rare LOC and generalized tonic-clonic seizure	Generalized tonic-clonic seizure	Automatism of hands, post-ictal amnesia
Anti-seizure medication history	Carbamazepine, valproate, clonazepam, lacosamide	Phenytoin, levetiracetam, lacosamide	Levetiracetam, lacosamide, topiramate	Zonisamide, levetiracetam, clonazepam	Topiramate, eslicarbazepine, lacosamide, clonazepam, levetiracetam, phenytoin	Lamotrigine, levetiracetam, zonisamide
Neuropsychologic testing	N/A	Cognition, language and memory performance average	Cognition language and memory performance average	Cognition and language performance below average	Cognition and memory performance below average	Average language performance, below average on non-verbal or higher-order executive function tasks
Preoperative imaging	MRI, fMRI	MRI, fMRI PET	MRI, fMRI	MRI, fMRI, PET	MRI, fMRI	MRI, fMRI, PET
Intracranial EEG localization	Hippocampal, anterior temporal cortex onset	Hippocampal, anterior temporal cortex onset	Hippocampal, anterior temporal cortex onset	Hippocampal, anterior temporal cortex onset	Hippocampal, anterior temporal cortex onset	Hippocampal, anterior temporal cortex onset
Resection location	Right anterior temporal lobectomy plus hippocampectomy	Left anterior temporal lobectomy plus hippocampectomy	Left anterior temporal lobectomy plus hippocampectomy	Right anterior temporal lobectomy plus hippocampectomy	Right anterior temporal lobectomy plus hippocampectomy	Right anterior temporal lobectomy plus hippocampectomy
Pathology findings	Mesial temporal sclerosis, microdysgenesis	Mesial temporal sclerosis, periventricular nodular heterotopia, microdysgenesis	Mesial temporal sclerosis, microdysgenesis	Mesial temporal sclerosis	Microdysgenesis	Mesial temporal sclerosis, microdysgenesis
Outcome (Engel) Score	1a	1a	1c	1b	1a	2a

fMRI = functional MRI; LOC = loss of consciousness; TBI = traumatic brain injury.

### Animal subjects

For *in vitro* experiments, primary cortical cultures were generated from either embryonic Day 18 (E18) (Ca^2+^ imaging) or postnatal Day 2 (P2) (MEA) Sprague-Dawley rats (Charles River Laboratories). Methods used to generate cultures for *in vitro* experiments were previously described and are included in the Supplementary material.^25,26^ For *in vivo* SUR1-TRPM4 expression and pharmacologic experiments, 6- to 8-week-old male and female C57/Bl6N mice were used (Charles River Laboratories). For constitutive *Trpm4* KO experiments, 12- to 16-week-old male and female C57/Bl6N + 129/SvJ mice were used.^27^ For neuron-specific *Trpm4* KO experiments, 6 to 8-week-old male and female animals were used; 8- to 12-week-old male and female C57/Bl6N + 129/SvJ mice were used for *Kcnj11* (KIR6.2) KO experiments.^28^ Genetic sequencing was performed commercially (Transnetyx) to confirm mouse genotypes prior to all KO experiments. Methods for generating neuron-specific Trpm4 KO mice and gene KO validation are described in the Supplementary material.

### Brain procurement

To procure brain samples, we euthanized and performed transcardial perfusion on mice first with ice-cold 0.9% normal saline. For western blot and quantitative PCR (qPCR) experiments, brains were then extracted and used. For immunofluorescence experiments, we followed saline perfusion with 4% PFA, then post-fixed brains at 4°C overnight in 4% PFA and submersed in 30% sucrose for 2–3 days. Following sucrose incubation, we cryopreserved samples in optimal cutting temperature (OCT) compound and generated 12 µm sections. We collected brain samples 2 days after the last PTZ administration for kindling experiments.

### PTZ kindling: standard and dose escalation

We used the PTZ kindling model to induce chronic epilepsy in mice and test differences in chronic seizure susceptibility between treatment groups. We used the standard model to assess SUR1-TRPM4 expressional differences in chronic epilepsy,^29^ while we used the dose-escalation approach to assess the effects of SUR1-TRPM4 inhibition on chronic seizure susceptibility as we recently described.^30^ Behavioural seizure responses were assessed 30 min after each injection for sham (SHAM) and PTZ animals by Racine scoring.^31^ The following classifications were used: 0: no seizure response; 1: behavioural arrest or slowing; 2. head nodding associated with facial clonus; 3: partial limb clonus with or without tail stiffening; 4: clonic seizure with rearing and loss of posture; 5: generalized tonic-clonic seizure with wild running and/or jumping; and 6: death. Methods used for continuous video EEG (vEEG) monitoring performed during PTZ kindling model characterization are described in the Supplementary material.

### Drug administration

We administered PTZ (Millipore-Sigma, P6500) to mice via intraperitoneal (IP) injection. For glyburide (GLYB) and 9-phenanthrol (9-PHEN) experiments, we reconstituted GLYB (Millipore-Sigma, G2539) or 9-PHEN (Millipore-Sigma 211281) in a corn oil vehicle (VEH) and administered 30 μg per mouse via IP injection daily and concurrently with PTZ treatments. On PTZ administration days, GLYB and 9-PHEN were given 15 min prior to PTZ injection.

### Immunofluorescence

For human tissue studies, we deparaffinized sections by a series of 10 min washes as follows: three washes in xylene, two in 100% ethanol, two in 95% ethanol, one in 70% ethanol, one in deionized water and one in 1× PBS. Cryopreserved mouse sections only underwent PBS washing to remove excess OCT. We performed antigen retrieval by incubating sections in Epitope Retrieval Solution (Cat. No. IW-1100; IHC World) using an Epitope Retrieval Steamer (Cat. No. IW-1102; IHC World) for 10 min at 100°C. After antigen retrieval, we washed sections then incubated in blocking buffer (0.2% Triton-X and 2% donkey serum in 1× PBS) for 1 h at room temperature (RT). We incubated sections in primary antibody resuspended in blocking buffer for 1–3 days at 4°C. After primary antibody incubation, we washed sections and incubated in species-appropriate fluorescent secondary antibodies (Alexa Fluor 488, 555 and 647 Molecular Probes, ThermoFisher Scientific) at a 1:500 dilution for 1 h at RT. We washed sections and cover-slipped using Prolong Gold Antifade mounting medium (Invitrogen, P36931). Primary antibody omissions were used as negative controls, and sections were immunolabelled as a single batch for each experiment.

### Image acquisition and quantification

We acquired images using a Nikon Ti2 epifluorescent microscope integrated with the Nikon Elements software (version 5.30.06) using a 20× objective and exposure time of 100–200 ms for all channels. All images for a given signal were captured using uniform parameters of magnification, area, exposure and gain. For unbiased human tissue SUR1-TRPM4 quantification, we randomly sampled 25 regions of interest (ROIs) across each tissue section. The number of cells with co-positive signals meeting a 2× signal intensity threshold for both SUR1 and TRPM4 were manually counted per field and averaged per section, then normalized to the control group average. For mouse tissue SUR1-TRPM4 quantification, we imaged single ROIs bilaterally within the CA3 region of the hippocampus and temporal cortex, then averaged quantified area fractions for SUR1 and TRPM4 meeting a 2× signal intensity threshold per channel per animal. We normalized quantified fractions for each signal to the SHAM group average. We characterized GFAP expression within the entire hippocampus and temporal cortex by generating whole-section 20× tiled images for each animal, defining the extent of each region using a mouse brain atlas, then quantifying the area fraction meeting a 2× signal intensity threshold within each region and averaging bilaterally per animal and normalizing to the SHAM group average. We performed cell-type localization analyses by measuring the Mander’s Overlap Coefficient (MOC) using the Just Another Co-localization Plugin^32^ with ImageJ^33^ to determine the extent of overlap between the neuron (NEUN+), astrocyte (GFAP+) or microglial (IBA1+) signal with either the SUR1 or TRPM4 signal (meeting 2× threshold). To determine which neuronal subtypes express SUR1-TRPM4, SUR1 and TRPM4 signals were qualitatively assessed in glutamatergic (CAMKIIA+) and GABAergic (GAD67+) cells.

### Co-immunoprecipitation and western blot

We compared SUR1-TRPM4 co-association between SHAM and PTZ-kindled mice brains using co-immunoprecipitation (co-IP) as previously described.^5^ After brain procurement, we enriched for regions containing the temporal cortex and hippocampus by cutting the brains coronally approximately 0.5 mm caudal to bregma and excluding the rostral portion. We homogenized samples in lysis buffer [1% Triton-X in 1/2× Dulbecco's PBS plus protease inhibitor (Roche Diagnostics, 11697498001)] and lysed cells for 30 min at 4°C. Following cell lysis, we centrifuged samples for 20 min at 14 000 G and collected the supernatant protein samples. We measured protein concentrations using the Bradford method (Bio-Rad, 5000006), and equal protein amounts were used for immunoprecipitation (IP). We performed TRPM4 IP by mixing a previously validated chicken primary TRPM4 antibody (5 µl, custom),^5^ IgY precipitating chicken resin (GenScript, L00405) and protein samples on a rotator overnight at 4°C. SUR1 IP was performed following the same procedure except with goat SUR1 antibody (5 µl, custom)^5^ and Protein G Dynabeads (ThermoFisher, 10003D). IPs using equal volumes of pre-immune sera generated during custom antibody development for each antibody were used as negative controls. Following IP, we washed protein samples then eluted by incubating washed resin in 2:1 NuPAGE Sample Reducing Agent (Invitrogen, NP0009) to NuPAGE lithium dodecyl sulfate (LDS) sample buffer (Invitrogen, NP0007) for 2 h at RT.

Following elution, we completely loaded IP samples and ran gel electrophoresis (Invitrogen, NP0036) in NuPAGE 3-(*N*-morpholino)propanesulfonic acid sodium dodecyl sulfate (MOPS SDS) Running Buffer (Invitrogen, NP0001-02) under a 160 V, 3 A applied current for 1 h 15 min. We transferred samples to 0.45 μm polyvinylidene fluoride (PVDF) membranes (Life Technologies, LC2005) for 1 h in 1× transfer buffer (NuPAGE Transfer Buffer, Invitrogen, NP00061 plus 10% methanol in diH_2_O). We incubated membranes in 5% milk for 5 min at RT, then primary antibodies were added, and membranes were incubated overnight at 4°C. Following primary antibody incubation, we washed membranes and incubated in secondary antibody in 1× tris-buffered saline with Tween 20 (TBST) for 1 h at RT. We developed protein signals using Atto development substrate (Thermo Scientific, A38556) with a luminescent imager (Fujifilm, LAS-3000). For co-IP experiments, after developing SUR1 signals, antibodies were stripped from membranes as described (Thermo Scientific, 21059) and reblotted for SUR1 or TRPM4. To quantify the degree of SUR1-TRPM4 co-association, densitometry western blot signals were analysed using ImageJ,^33^ and detected SUR1 signals were normalized to TRPM4 IP signals.

### Low magnesium (+ TRPM4 inhibitor) treatments

We used a low Mg^2+^  *in vitro* model of neuronal activation to induce seizure-like neuron hyperactivation, as we previously described.^25^ For low Mg^2+^ treatments, we incubated cells for 2 h in either control or low Mg^2+^ artificial CSF (aCSF) [control aCSF: 10 mM 4-(2-hydroxyethyl)-1-piperazineethanesulfonic acid (HEPES) solution, 2.5 mM KCl, 2 mM CaCl_2_, 2 mM glycine, 10 mM glucose, 145 mM NaCl and 1 mM MgCl_2_ in deionized water, pH 7.3; low Mg^2+^: same as control except with 0 mM MgCl_2_]. For co-treatment conditions, we added either VEH (0.01% dimethyl sulfoxide), 4-chloro-2-[2-(2-chloro-phenoxy)-acetylamino]-benzoic acid (CBA) (10 µM) or 4-chloro-2-(2-(naphthalene-1-yloxy) acetamido) benzoic acid (NBA) (10 µM) to low Mg^2+^ aCSF solutions. After 2 h, we removed treatment aCSF and returned cells to maintenance media.

### MEA analysis

We recorded rat cortical cultures on 96-well MEA plates containing eight electrodes that record extracellular voltage with a sampling rate of 12.5 kHz using a Maestro Pro MEA system (Axion BioSystems). We used the Neural Metrics Tool (Axion BioSystems) to detect neuronal action potentials (spikes), defined as when the recorded trace exceeded a threshold of ±6 standard deviations from the baseline signal, and bursts, defined as occurrences of five spikes on a single electrode with a maximal inter-spike interval of 100 ms. We monitored baseline activity of all cultures from 10 days *in vitro* (DIV10) to DIV17, and only culture wells demonstrating sufficient and stable firing, defined by a mean firing rate of at least 6 Hz, were used. On each treatment day, we recorded population activity for 5 min before and after treatments to obtain baseline and post-treatment measurements, then computed the rate of neuronal bursts in every electrode in each well and averaged per condition for pre- and post-treatment recordings. This generated pre-treatment and post-treatment burst frequency values for each treatment per day. From these values, we calculated the burst frequency ratio (BFR) for each group within each day defined as the post-treatment burst frequency/pre-treatment burst frequency.

### Ca^2+^ imaging and analysis

To assess neuronal Ca^2+^ responses, we loaded neurons with a Rhod-2 AM dye (Invitrogen, R1244) as recommended by the manufacturer. We imaged Ca^2+^ responses in neurons expressing green fluorescent protein (GFP) signals for somatic Ca^2+^ responses using a W1 Nikon confocal microscope with a 20× objective at 37°C. We treated neurons with control aCSF for 1 min to obtain a baseline Ca^2+^ response, then with low Mg^2+^ aCSF for 9 min to induce hyperactivity. To quantify Ca^2+^ responses, we calculated ΔF/F0 values and plotted for each frame [(Fframe–F0)/F0] in GFP+ neurons to generate overall response curves for each cell. We quantified the area under the curve (AUC) and maximal response of each cell using a custom MATLAB script.

### Statistical analysis

Data are presented as mean ± standard error of the mean unless otherwise noted. Student’s *t*-test, two-way ANOVA with Tukey’s correction, or non-linear regression analyses with extra sum-of-squares F test were used as appropriate and identified in the figure legends. One-tailed *t*-tests were used when assessing SUR1-TRPM4 expression, as control expression is expected to be minimal based on substantial prior literature,^4-6,9,10,12,34^ and in our identification of PTZ susceptible brain regions marked by GFAP upregulation. Two-tailed analyses were used for all other comparisons of two groups. All statistical values are reported in the figure legends. Analyses were performed with GraphPad Prism 9.3.1. *P* < 0.05 was deemed to be statistically significant.

## Results

### SUR1-TRPM4 protein expression in human epileptic tissue

**Figure 1:**
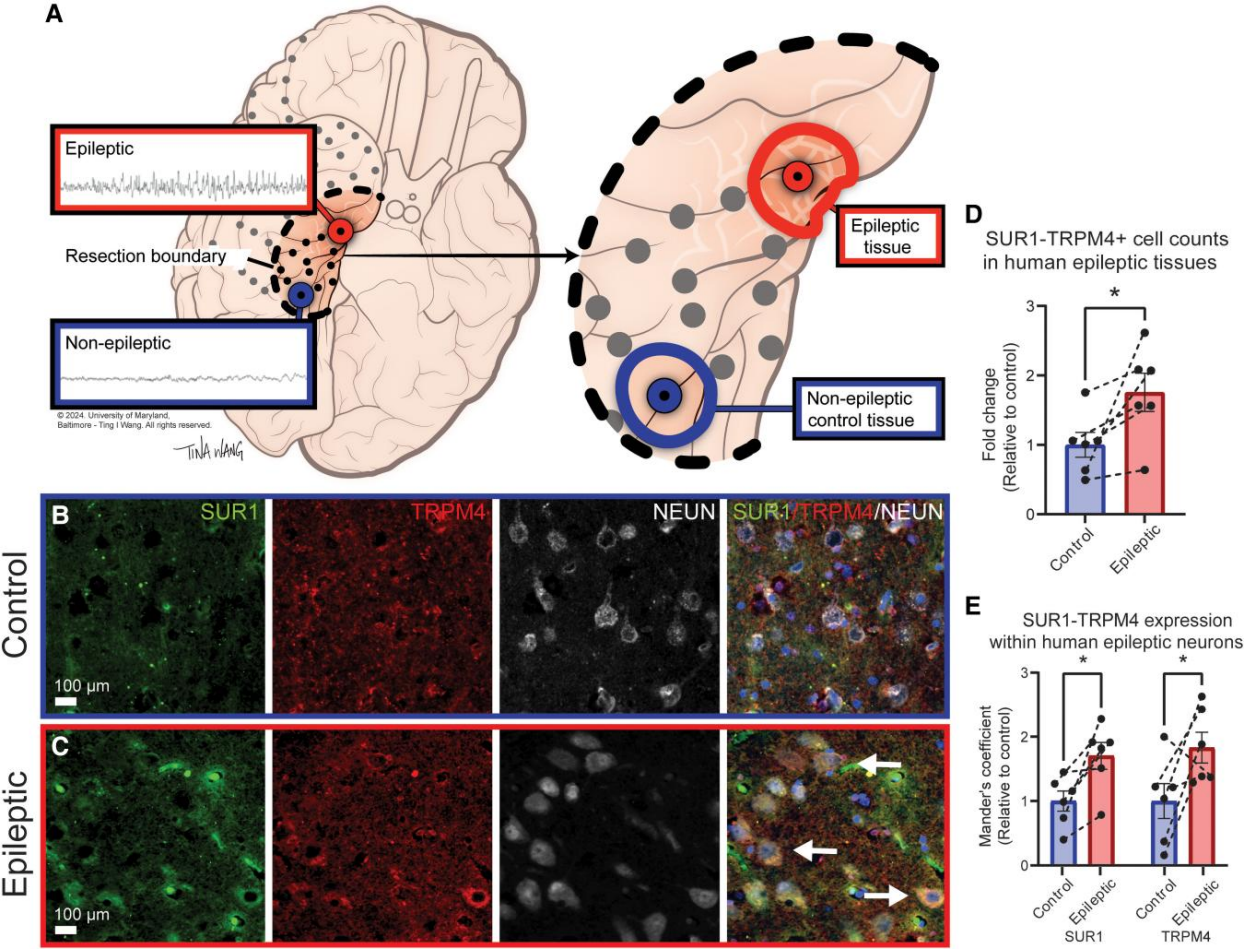
**SUR1-TRPM4 is overexpressed in neurons within human epileptic tissues**. (**A**) Theoretical intracranial electrode grid placement and tissue sorting method. Regions within the resection boundary are electrographically identified as epileptic (red) or non-epileptic (blue), then after resection are separated into epileptic and control tissue samples. Voltage traces of seizure versus non-seizure recordings from two separate electrodes. (**B** and **C**) Immunolabelled tissues from epilepsy patients in control (**B**) and epileptic (**C**) samples. Epileptic zones demonstrate increased SUR1 (green) and TRPM4 (red) signals within NEUN+ cells (white) compared with control regions. (**D**) Quantifications of SUR1-TRPM4 co-expressing cell counts show elevated co-expression in epileptic zones (*P =* 0.02, *t =* 2.31, df = 10). (**E**) Correlation between SUR1-TRPM4 and NEUN signals (neurons) were elevated in epileptic tissue compared with control regions (SUR1: *P =* 0.01, *t =* 3.47, df = 5; TRPM4: *P =* 0.03, *t =* 2.32, df = 5). Data are represented as mean ± standard error of the mean. Full ×20 magnification immunofluorescent images are available in Supplementary Fig. 1; *n =* 6 patients, *P < 0.05, one-tailed paired *t*-test. Figure 1A credit to Tina I. Wang, all rights reserved.

We first assessed SUR1-TRPM4 protein expression in temporal cortex or hippocampal tissues resected from chronic drug-resistant epilepsy patients with mesial temporal sclerosis (Table 1). All patients achieved near-seizure freedom after surgical resection (Table 1). Using immunofluorescent labelling for SUR1 and TRPM4, we analysed tissues from six patients electrographically sorted within the same patients as either normal (control) or epileptic (Fig. 1A–C and Supplementary Fig. 1). Epileptic brain samples exhibited significantly more SUR1-TRPM4 co-positive cells compared with control tissue (Fig. 1B–D) (1.76 ± 0.27 fold-change). Co-labelling with the neuronal marker NEUN revealed significantly elevated MOC for SUR1 (1.70 ± 0.21 fold-change) and TRPM4 (1.83 ± 0.24 fold-change) in epileptic neurons compared with controls (Fig. 1E). These findings demonstrate that SUR1-TRPM4 expression is upregulated in neurons within seizing regions of the human epileptic brain.

### SUR1-TRPM4 expression and co-association after chronic seizures in mice

**Figure 2:**
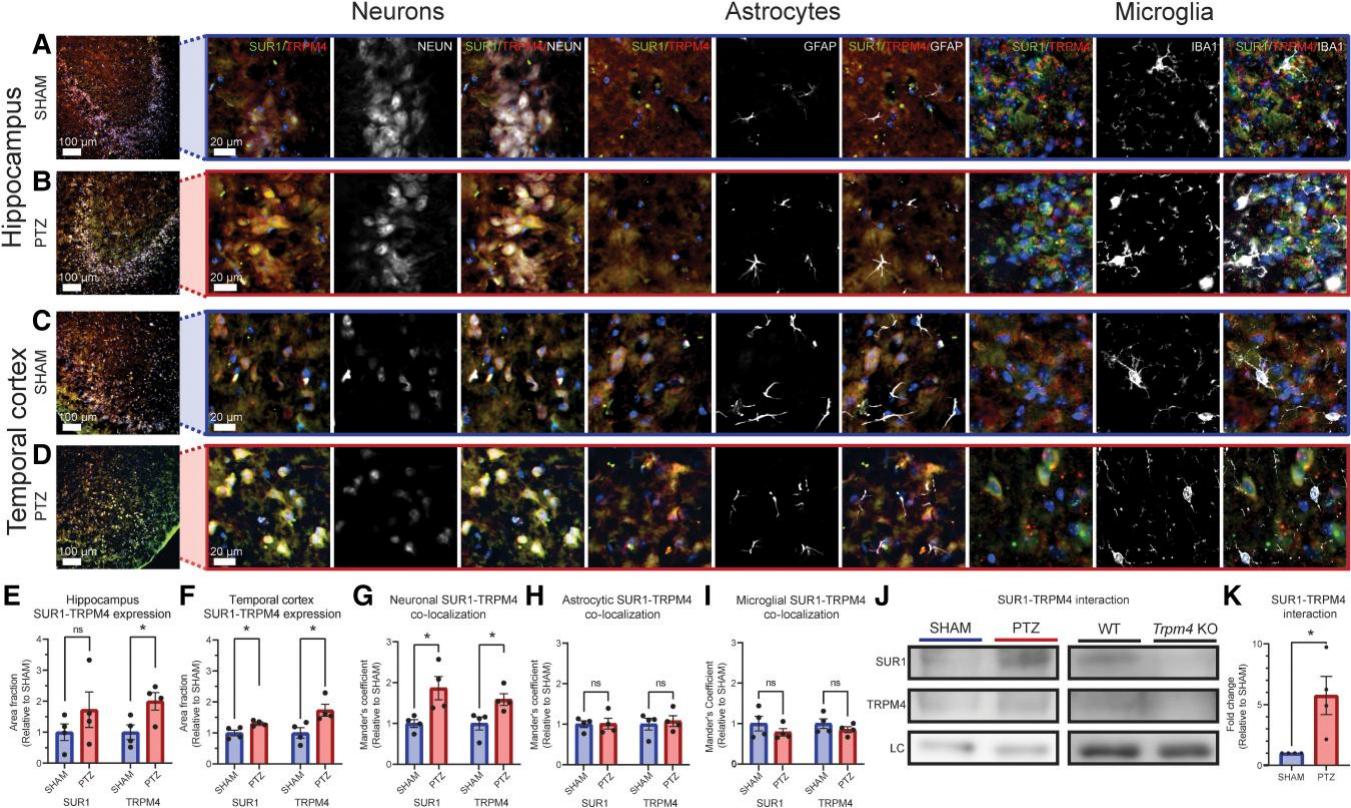
**The SUR1-TRPM4 channel is upregulated and co-associated in a mouse PTZ kindling chronic epilepsy model**. (**A–D**) Immunofluorescent images of SUR1-TRPM4 within the hippocampus and temporal cortex of SHAM and PTZ-kindled mice. Both SUR1 (green) and TRPM4 (red) are elevated in PTZ samples compared with SHAM, and signals localize to NEUN+ cells (white) more than GFAP+ or IBA1+ cells (white). (**E** and **F**) Quantification of SUR1-TRPM4 expression within the hippocampus (**E**) and temporal cortex (**F**) of SHAM and PTZ mice. SUR1 was significantly increased (*P =* 0.01, *t =* 3.02, df = 6) in the PTZ-kindled temporal cortex and non-significantly (*P =* 0.15, *t =* 1.15, df = 6) elevated in the hippocampus. TRPM4 expression was significantly elevated within the temporal cortex (*P =* 0.01, *t =* 2.90, df = 6) and hippocampus (*P =* 0.02, *t =* 2.70, df = 6) in PTZ-kindled mice. (**G–I**) PTZ-kindled mice, compared with SHAM, demonstrated increased correlations for both SUR1 and TRPM4 signals with NEUN+ cells; (**G**) (SUR1: *P =* 0.01, *t =* 2.88, df *=* 6; TRPM4: *P =* 0.02, *t =* 2.72, df = 6), but not with GFAP+, (**H**) (SUR1: *P =* 0.47, *t =* 0.07, df = 6; TRPM4: *P =* 0.35, *t =* 0.39, df = 6) or IBA1+ cells, (**I**) (SUR1: *P =* 0.16, *t =* 1.06, df *=* 6; TRPM4: *P =* 0.16, *t =* 1.07, df = 6). (**J**) Representative western blots for SUR1-TRPM4 co-IP from SHAM versus PTZ mice brains and PTZ-kindled WT versus *Trpm4* KO mice. SUR1 and TRPM4 blots detect a higher SUR1 signal in PTZ mice with equal TRPM4 IP and equal loading controls (LC) compared with SHAM. (**K**) Quantification for SUR1-TRPM4 co-IP demonstrates elevated co-association (*P =* 0.01, *t =* 3.02, df *=* 6) between SUR1 and TRPM4 in the PTZ brain compared with SHAM. Signals for SUR1 co-IP are normalized to the total TRPM4 immunoprecipitated for each sample. Data are represented as mean ± SEM. Full ×20 magnification immunofluorescent images are available in Supplementary Figs 3 and 4 and full blots for co-IP data are available in Supplementary Fig. 6; *n =* 4 mice per group for each assay, *P < 0.05, one-tailed unpaired *t*-tests. KO = knockout; PTZ = pentylenetetrazol, IP = immunoprecipitation, WT = wild-type.

**Figure 3:**
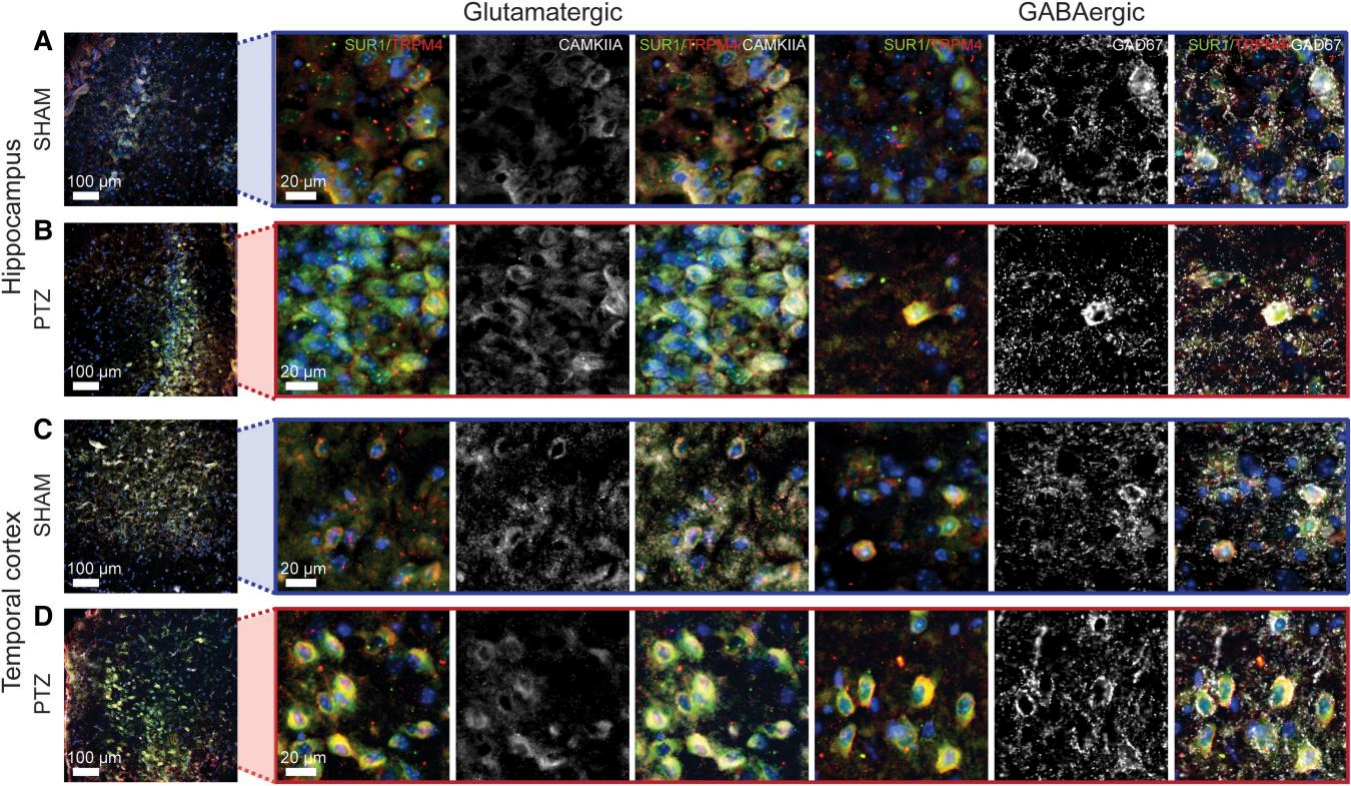
**SUR1-TRPM4 is expressed by excitatory and inhibitory neurons**. (**A–D**) Immunofluorescent images of SUR1-TRPM4 within the hippocampus (**A** and **B**) and temporal cortex (**C** and **D**) of SHAM (**A** and **C**) and PTZ-kindled (**B** and **D**) mice co-labelled with markers (white) for glutamatergic (CAMKIIA) and GABAergic (GAD67) neurons. Signals for SUR1 (green) and TRPM4 (red) were observed in both neuronal subtypes. Full ×20 magnification immunofluorescent images are available in Supplementary Fig. 5 (qualitative results are representative for *n =* 4 mice per group). PTZ = pentylenetetrazol.

We next examined whether SUR1-TRPM4 expression is elevated following chronic seizures in mice, independent of any initial insult triggering epileptogenesis (e.g. SE), using the PTZ kindling model. The PTZ kindling model shares several key characteristics with human temporal lobe epilepsy that make it a reliable and relevant model to study epileptogenesis, including the chronic lowering of seizure threshold and key neuropathologic changes such as mossy fibre sprouting, neuronal loss, and gliosis in the hippocampus and temporal cortex.^35,36^ Furthermore, compared with other chemoconvulsant chronic seizure models such as lithium-pilocarpine and intrahippocampal kainate, which require induction of SE to generate chronic seizures,^37,38^ PTZ kindling is the ideal model to investigate chronic protein expressional alterations resulting from chronic seizures separately from those caused by SE because PTZ kindling does not require the induction of SE to achieve chronic seizures. As previously described,^29^ PTZ kindling with chronic sub-convulsive PTZ doses (35 mg/kg) induced progressively worsening seizures, confirmed behaviourally and electrographically, with preferential involvement of the hippocampus and temporal cortex (Supplementary Fig. 2). No seizure activity was observed in SHAM control animals. To identify brain regions preferentially affected in our model, we immunolabelled for GFAP, a reactive astrocytosis marker previously shown to be upregulated in the temporal cortex and hippocampus in PTZ animals.^39-41^ We observed significant GFAP upregulation in the hippocampus (2.05 ± 0.57 fold-increase) and temporal cortical regions including the piriform, perirhinal and entorhinal cortices (3.06 ± 0.57 fold-increase) in PTZ-kindled mice compared with SHAM controls (Supplementary Fig. 2G–I). These brain regions correspond to locations of resected tissues obtained from our epilepsy patient cohort (Fig. 1 and Table 1). Immunofluorescent labelling within regions affected by PTZ kindling (Fig. 2 and Supplementary Figs 3 and 4) revealed significantly upregulated SUR1 in the temporal cortex (1.31 ± 0.04 fold-change), and a trend towards significance in the hippocampus (1.73 ± 0.57 fold-change) of PTZ-kindled mice compared with SHAM (Fig. 2E and F). TRPM4 expression was significantly increased in both the hippocampus (2.00 ± 0.28 fold-change) and the temporal cortex (1.73 ± 0.19 fold-change) (Fig. 2E and F). Co-localization analyses showed significant increases in MOC between SUR1 (1.87 ± 0.28 fold-change) or TRPM4 (1.58 ± 0.29 fold-change) and NEUN, but not GFAP or IBA1 for either SUR1 (GFAP: 1.01 ± 0.13 fold-change, IBA1: 0.79 ± 0.09 fold-change) or TRPM4 (GFAP: 1.08 ± 0.13 fold-change, IBA1: 0.85 ± 0.14 fold-change) (Fig. 2G–I and Supplementary Figs 3 and 4), suggesting neuronal-predominant upregulation of SUR1-TRPM4 channel expression. Further neuronal subtype assessment demonstrated that both excitatory (CAMKIIA+) and inhibitory (GAD67+) neurons express SUR1-TRPM4 (Fig. 3 and Supplementary Fig. 5).

Building on the observed SUR1-TRPM4 co-expression via immunofluorescence, we investigated whether this co-expression translates into increased SUR1-TRPM4 channel formation in the epileptic brain. Since SUR1 binding to TRPM4 enhances its open probability 30-fold under low ATP conditions,^5^ such co-association could amplify the channel’s pro-excitatory effects in epilepsy. To assess this, we performed co-IP on hippocampal and temporal cortical regions from SHAM and PTZ-kindled mice (Fig. 2J and K and Supplementary Fig. 6). Within brain samples enriched for GFAP-positive, injured brain regions, we demonstrated a significant increase in SUR1-TRPM4 co-association in PTZ-kindled brains compared with SHAM controls (5.76 ± 1.58 fold-change). Validation using TRPM4 KO mice confirmed the specificity of this co-association (Fig. 2J and Supplementary Fig. 6D–F). These results indicate that increased SUR1 and TRPM4 expression corresponds to enhanced SUR1-TRPM4 channel formation in our epilepsy model.

### *In vitro* SUR1-TRPM4 inhibition is protective against seizure-like activity

**Figure 4:**
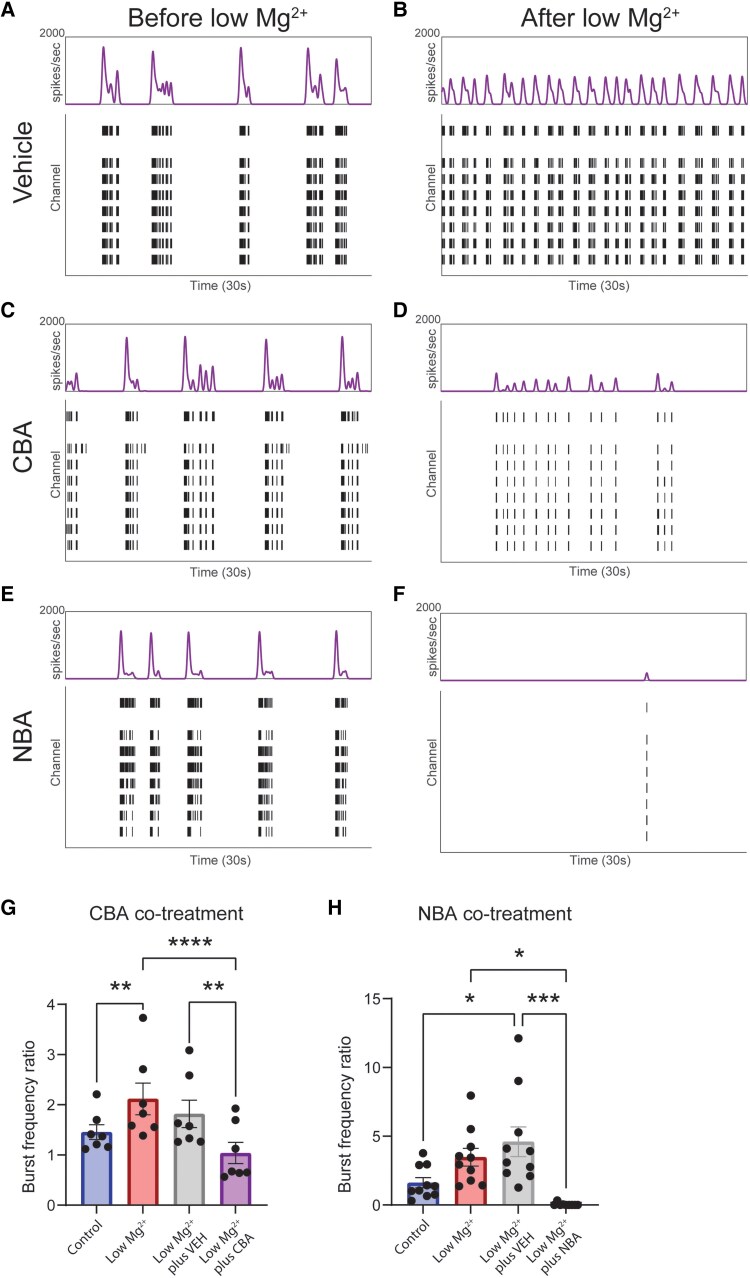
**TRPM4 inhibition *in vitro* reduces hyperexcitation evoked by low Mg^2+^**. (**A–F**) Representative real-time smoothed spike frequency histograms (*top*) and raster plots (*bottom*) captured with MEA for TRPM4 inhibitor (CBA, NBA) experiments. Plots are shown for VEH, CBA and NBA co-treatments with low Mg^2+^ before and after low Mg^2+^ treatment. (**G** and **H**) Quantification of neuron population burst responses to control aCSF, untreated low Mg^2+^ aCSF and low Mg^2+^ co-treated with VEH, CBA or NBA. As expected, low Mg^2+^ increases bursting compared with control (*P =* 0.005, *F =* 15.42, df = 18),^25^ and VEH co-treatment with low Mg^2+^ did not significantly affect this response (*P =* 0.31, *F =* 15.42, df = 18). Co-treatment with CBA (untreated low Mg^2+^: *P <* 0.0001, *F =* 15.42, df = 18; VEH co-treated*: P =* 0.001, *F =* 15.42, df *=* 18) or NBA (untreated low Mg^2+^: *P =* 0.02, *F =* 7.82, df = 26; VEH co-treated: *P =* 0.001, *F =* 7.82, df = 26) significantly attenuated this response. Data are represented as mean ± standard error of the mean. (CBA experiment: *n = 7* treatments per group, NBA experiment: *n =* 10 treatments per group; *P < 0.05, **P < 0.01, ***P < 0.001, ****P < 0.0001, two-way ANOVA with Tukey’s *post hoc* correction). One treatment day was excluded for the low Mg^2+^+NBA group. aCSF = artificial CSF; MEA = microelectrode array; VEH = vehicle.

To determine the functional role of SUR1-TRPM4 in seizure-like elevations in neuron activity, we tested two selective TRPM4 inhibitors, CBA (10 µM) or NBA (10 µM), in a low Mg^2+^  *in vitro* model of hyperactivity. TRPM4, the molecular target for CBA and NBA and the pore-forming component of the SUR1-TRPM4 channel, is well expressed in our *in vitro* low Mg^2+^ model (Supplementary Fig. 7). As previously described, low Mg^2+^ treatment significantly increased hyperactivity, as indicated by elevated BFRs (control BFR: 1.45 ± 0.15, low Mg^2+^: 2.12 ± 0.32) (Fig. 4A and B) across multiple days. This mimics the effects of PTZ kindling observed *in vivo* via a different mechanism.^4,25^ Daily co-treatment of low Mg²⁺ aCSF with either CBA or NBA substantially attenuated hyperactivity. CBA co-treatment significantly reduced BFR (1.04 ± 0.21) compared with either untreated (BFR: 1.45 ± 0.15) or VEH co-treated (BFR: 1.81 ± 0.27) low Mg^2+^ conditions (Fig. 4C and D). NBA co-treatment nearly abolished bursting (BFR: 0.04 ± 0.04) compared with untreated (BFR: 3.48 ± 0.65) or VEH-treated (BFR: 4.60 ± 1.08) conditions (Fig. 4E and F). These findings demonstrate that TRPM4 inhibition reduces seizure-like activity induced by low Mg²⁺ (Fig. 4G and H) and suggest that SUR1-TRPM4 plays a critical role in neuronal hyperexcitability underlying seizures.

### *In vitro* SUR1-TRPM4 overexpression increases neuron hyperexcitation

**Figure 5:**
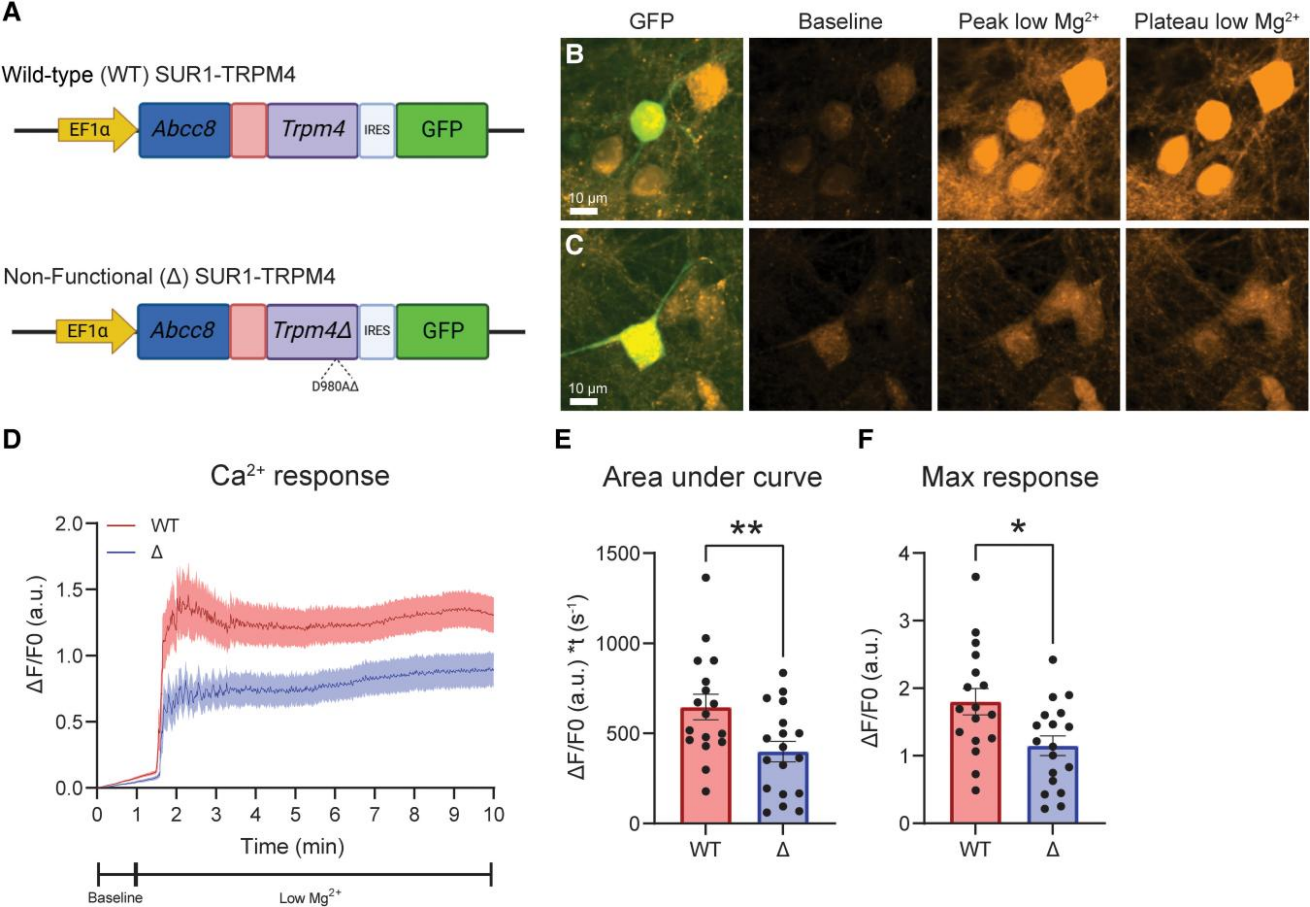
**SUR1-TRPM4 overexpression in neurons elevates neuronal response to low Mg^2+^ stimulation**. (**A**) Schematics for SUR1-TRPM4 overexpression plasmids. Overexpression of functional (WT) SUR1-TRPM4 was compared with overexpression of a non-functional SUR1-TRPM4 (Δ) containing a single point mutation (D980A) in the pore forming portion of the TRPM4 channel.^42^ (**B** and **C**) Representative images for cell identification (GFP) and Rhod-2 Ca^2+^ signals prior to low Mg^2+^ exposure (baseline), at the time of peak Rhod-2 response to low Mg^2+^ (minute 2 of recording), and during the end plateau phase (minute 9). Note the greater intensity change in WT transfected neurons compared with Δ after low Mg^2+^ exposure. (**D**) Ca^2+^ response curves averaged across all WT (red) and Δ (blue) SUR1-TRPM4 transfected neurons. The first minute of the response occurred in baseline conditions, then the final 9 min in low Mg^2+^ conditions. Note the WT trace is elevated across the low Mg^2+^ period compared with the Δ trace. (**E** and **F**) Quantifications of the AUC (*P =* 0.01, *t =* 2.74, df = 33) and maximum response (*P =* 0.01, *t =* 2.68, df = 33) for individual neurons overexpressing WT or Δ SUR1-TRPM4. Both metrics show significantly greater Ca^2+^ responses in WT transfected cells compared with Δ. Data are represented as mean ± standard error of the mean. WT: *n =* 17 cells, Δ *n =* 18 cells; *P < 0.05, **P < 0.01; two-tailed unpaired *t*-tests. AUC = area under the curve; WT = wild-type. Figure 5A was created in BioRender by Moyer, M. (2025) (https://BioRender.com/66c9sgj).

To further investigate the impact of SUR1-TRPM4 overexpression on neuronal hyperexcitability, we transfected neurons with a functional SUR1-TRPM4 fusion protein (wild-type, WT) or a non-functional control (Δ) with a single point mutation (D980A) in the pore-forming region of the *Trpm4* sequence as previously reported,^42^ and we measured Ca^2+^ responses during low Mg^2+^ stimulation (Fig. 5A). Prior to experimentation, plasmid sequences were confirmed (UMB Genomics Core), and successful expressions of both WT and Δ SUR1-TRPM4 were validated (Supplementary Fig. 8). To image Ca^2+^ responses, transfected cells were loaded with Rhod-2 dye 30 min prior to experiments, and successfully transfected cells were identified by bicistronic expression of a green fluorescent protein reporter (GFP) (Fig. 5B and C). Cells were imaged for 1 min continually perfused with control aCSF, then for 9 min with continual low Mg^2+^ aCSF perfusion (Fig. 5D). Compared with the Δ control, neurons expressing WT SUR1-TRPM4 channels demonstrated a greater Ca^2+^ response to low Mg^2+^ stimulation compared with non-functional controls (Fig. 5D), as demonstrated by an increased AUC [WT: 646.17 ± 70.47 (arbitrary units, a.u.) × t(s^−1^); Δ: 398.97 ± 57.07 (a.u.) × t(s^−1^)] (Fig. 5E) and peak response [WT: 1.80 ± 0.20 (a.u.); Δ: 1.15 ± 0.15 (a.u.)] (Fig. 5F). Together, these data suggest that SUR1-TRPM4 overexpression enhances neuronal hyperexcitability induced by low Mg^2+^.

### *In vivo* SUR1-TRPM4 inhibition is protective against chronic seizure development

**Figure 6:**
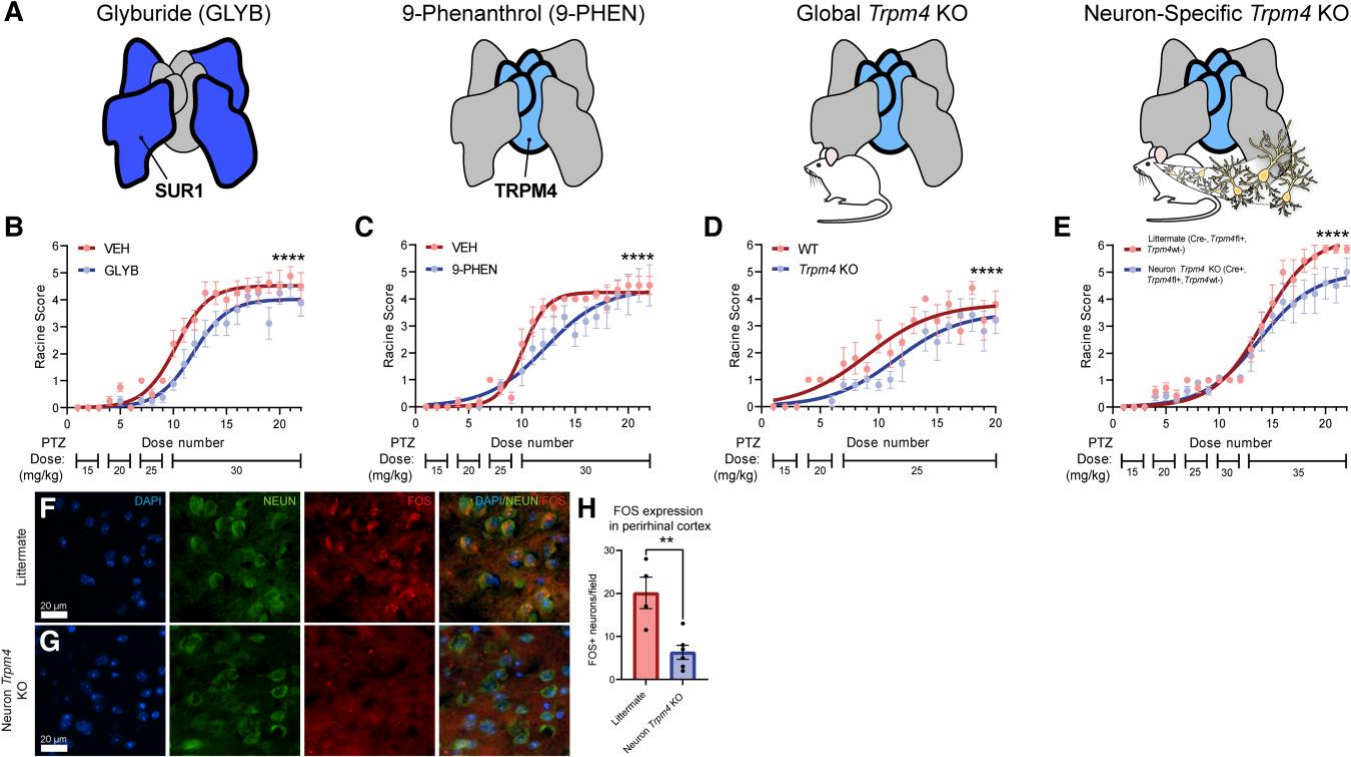
**SUR1-TRPM4 inhibition *in vivo* attenuates PTZ-kindling-induced chronic seizure development by reducing neuronal activation**. (**A**) Methods employed for SUR1-TRPM4 inhibition in PTZ kindling. GLYB is a pharmacologic inhibitor for the SUR1 component of the channel, whereas 9-PHEN inhibits TRPM4. Constitutive and neuron-specific *Trpm4* KO experiments were conducted to elucidate the effect of SUR1-TRPM4 inhibition specifically in neurons. (**B–E**) SUR1-TRPM4 inhibition either by pharmacological SUR1 inhibition (VEH: *n =* 8 mice, GLYB: *n =* 8 mice), pharmacologic TRPM4 inhibition (VEH: *n =* 6 mice, 9-PHEN: *n =* 6 mice), constitutive *Trpm4* KO (WT: *n =* 5 mice, KO: *n =* 5 mice), or neuron-specific conditional *Trpm4* KO (WT: *n =* 7 mice, KO: *n =* 10 mice) during PTZ kindling significantly reduces (*P <* 0.0001 for all) chronic seizure development, indicated by decreased Racine scores, compared with their respective controls. (**F** and **G**) Representative immunofluorescent images after labelling for DAPI (blue), NEUN (green), FOS (red) and combined channels taken within the perirhinal cortex of WT littermate (**F**) compared with neuron-specific *Trpm4* KO (**G**) mice within the perirhinal cortex after PTZ kindling (WT: *n =* 4 mice, KO: *n =* 6 mice). Note more NEUN+ cells exhibit co-localized FOS expression in WT than *Trpm4* KO conditions. (**H**) Quantifications of co-positive cells for NEUN and FOS demonstrated significantly greater (*P =* 0.005, *t =* 3.90, df = 8) FOS expression in WT mice than in neuron-specific *Trpm4* KO mice. Data are represented as mean ± standard error of the mean. **P < 0.01, ****P < 0.0001, logistic growth non-linear regression analysis for Racine score data, unpaired two-tailed *t*-test for FOS expression data. KO = knockout; PTZ = pentylenetetrazol; VEH = vehicle; WT = wild-type. Figure 6A credit to Tina I. Wang, all rights reserved.

We inhibited SUR1-TRPM4 within a dose-escalation PTZ kindling model, an approach we recently developed to enhance sensitivity for detecting differences in seizure susceptibility,^30^ to determine SUR1-TRPM4 involvement in reducing the chronic seizure threshold. The SUR1-TRPM4 channel was inhibited concurrently with PTZ kindling using several strategies: pharmacologic SUR1 inhibition with GLYB, TRPM4 inhibition with 9-PHEN, global *Trpm4* genetic KO and neuron-specific *Trpm4* conditional KO (Fig. 6A). GLYB and 9-PHEN were selected based on their established use in *in vivo* studies, with GLYB’s application in CNS injury being well characterized.^4,7,8^ Additionally, GLYB is an FDA-approved drug for type II diabetes and is currently undergoing clinical trials for stroke and traumatic brain injury (TBI).^7,8^ Constitutive KO of *Trpm4*, the pore forming and thus the functional component of the SUR1-TRPM4 channel, validated target specificity of pharmacologic inhibition, while neuron-specific *Trpm4* KO tested the hypothesis that neuronal SUR1-TRPM4 expression contributes to worsened seizure susceptibility. Successful *Trpm4* KO was confirmed before experimentation (Supplementary Fig. 9). To exclude effects from KIR6.2 inhibition, an alternative SUR1-regulated channel under normal conditions,^4^ we also assessed constitutive *Kcnj11* (KIR6.2) KO in PTZ kindling (Supplementary Fig. 10).

In all conditions, SUR1-TRPM4 inhibition significantly attenuated chronic seizure responses to PTZ kindling, as evidenced by rightward shifts and/or decreased maximal plateau in behavioural scoring curves (Fig. 6B–E). In contrast, *Kcnj11* KO had no effect on chronic seizure response (Supplementary Fig. 10A). Together, these data suggest that inhibiting SUR1 or TRPM4, but not KIR6.2, reduces chronic seizure susceptibility confirming the role of the SUR1-TRPM4 channel in epilepsy. Furthermore, *Trpm4* KO results validate the specificity of pharmacologic inhibition and support neuronal SUR1-TRPM4 expression as a key pro-seizure mechanism.

Finally, to assess whether the anti-seizure effects of SUR1-TRPM4 inhibition resulted from reduced neuronal activation, we performed FOS protein immunofluorescence in neuron-specific *Trpm4* KO and littermate control mice (Fig. 6F–H).^43,44^ Neuronal FOS expression was significantly reduced within the perirhinal cortex (a critical region for hippocampal seizure propagation in PTZ kindling)^45^ in KO mice (6.33 ± 1.63 FOS+ cells/field) compared with littermate controls (20.13 ± 3.67 FOS+ cells/field). These findings suggest that SUR1-TRPM4 inhibition reduces PTZ-induced neuronal hyperexcitation in key temporal cortical regions involved in seizures.

## Discussion

This study is the first to characterize the role of neuronal SUR1-TRPM4 expression in driving hyperactivity and seizures in chronic epilepsy independent of SE. We found that SUR1-TRPM4 expression is upregulated in neurons in both human epileptic tissue (Fig. 1) and a rodent PTZ kindling model (Figs 2 and 3). Furthermore, we showed that inhibiting SUR1-TRPM4 reduces neuronal population hyperexcitation and chronic seizure susceptibility (Figs 4 and 6), while overexpression increases neuronal excitability (Fig. 5). These findings suggest that SUR1-TRPM4 could serve as both a biomarker of epileptic tissue and a therapeutic target for seizure management.

Our findings build upon prior research investigating SUR1-TRPM4 upregulation in epilepsy. Consistent with our results, increased SUR1-TRPM4 expression has been observed for at least 7 days in SE-based chronic epilepsy models, including lithium-pilocarpine and intrahippocampal kainate.^20-22^ SUR1-TRPM4 inhibition in these models via GLYB or constitutive KO reduced spontaneous chronic seizures after SE.^20-22^ Our study extends these findings towards non-SE chronic epilepsy, an important distinction as SE is the initial aetiology in only a small minority of epilepsy patients,^46^ by demonstrating anti-seizure effects of SUR1-TRPM4 inhibition in PTZ kindling (Fig. 6). We also provide evidence for a novel mechanism through which neuronal SUR1-TRPM4 expression promotes chronic seizures by increasing neuronal hyperexcitability, as shown by enhanced SUR1-TRPM4 co-association (Fig. 2 and Supplementary Fig. 6), amplified excitability by neuronal SUR1-TRPM4 overexpression (Fig. 5), and reduced hyperactivity (Fig. 4) and seizure susceptibility (Fig. 6) with neuronal SUR1-TRPM4 inhibition. Previous patch clamp studies showed TRPM4’s role as a key contributor to intrinsic neuronal excitability and bursting,^17,18,47,48^ and our work expands on this understanding by demonstrating TRPM4’s impact on population-level hyperactivity (Fig. 4), a key driver of seizures.^49^

**Figure 7:**
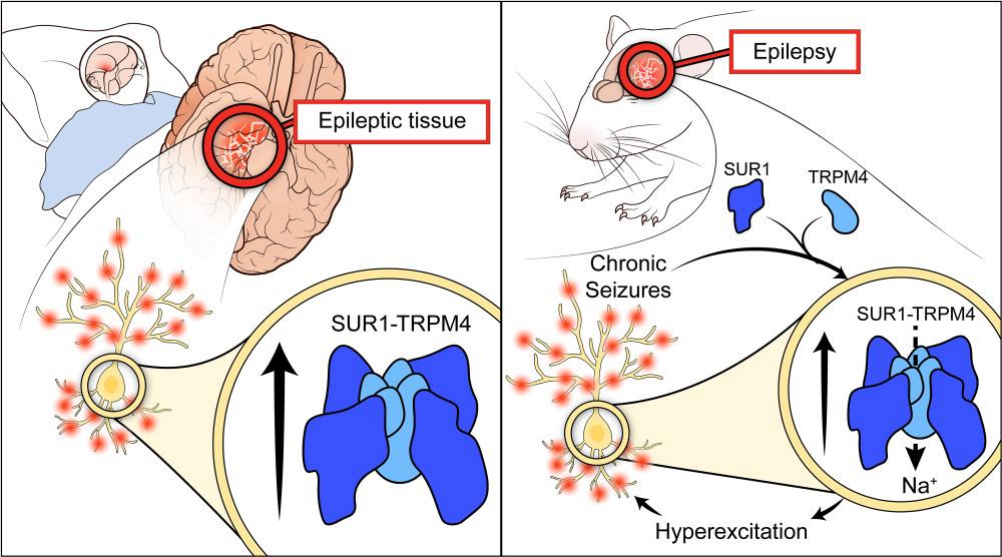
**SUR1-TRPM4 expression in neurons promotes hyperexcitability and seizures in epilepsy**. Figure 7 credit to Tina I. Wang, all rights reserved.

Based on prior literature and our data, we propose a reciprocal relationship between SUR1-TRPM4 expression and seizure susceptibility (Fig. 7). SUR1-TRPM4 expression is transcriptionally regulated through neuroinflammatory [nuclear factor kappa-light-chain-enhancer of activated B cells (NF-κB)] and metabolic [specificity protein 1 (Sp1) and hypoxia inducible factora 1α (HIF1α)] pathways,^50^ which are both persistently elevated in epileptic tissue.^51,52^ Chronic seizures in PTZ kindling likely activate these pathways, driving SUR1-TRPM4 upregulation, which further propagates seizure susceptibility in a feed-forward manner through neuronal excitation. SUR1-TRPM4 is a depolarizing ion channel when activated^4,53^; therefore, its expression in epileptic neurons increase neuronal activity by directly depolarizing membrane potential. Additionally, we recently demonstrated that SUR1-TRPM4 activation in astrocytes after stroke leads to Ca^2+^ influx, through a functional interplay with sodium-calcium exchangers (NCX).^54^ This could also occur in epileptic neurons, where NCXs are constitutively expressed, which may also affect neuron activity by promoting synaptic neurotransmitter release and/or causing differential regulation of gene expressions. Our data also suggest that neuronal SUR1-TRPM4 overexpression independently increases excitability under hyperexcitable conditions *in vitro* (Fig. 5). Inhibition of SUR1-TRPM4 during PTZ kindling reduced both seizure susceptibility and neuronal activation, as indicated by decreased FOS expression (Fig. 6), laying out a potential mechanism through which SUR1-TRPM4 promotes seizures. Epilepsy is characterized by increased overall seizure network excitability driven by elevated glutamatergic neuron activity relative to total GABAergic neuron inhibitory input. This occurs due to several mechanisms, including altered ion channel expression/function, selective loss of GABAergic neurons, and synaptic plasticity and sprouting leading to network reorganization.^55,56^ Interestingly, we observed SUR1-TRPM4 expression in both excitatory and inhibitory neurons (Fig. 3 and Supplementary Fig. 5). When taken together with the anti-convulsive effect of SUR1-TRPM4 inhibition, this suggests that any inhibitory effect driven by SUR1-TRPM4 activity in GABAergic neurons is outweighed by the increased excitation driven by SUR1-TRPM4 expression in glutamatergic neurons. This is especially supported by our neuron-specific *Trpm4* KO findings (Fig. 6E), in which KO was driven under a calcium/calmodulin-dependent protein kinase IIa (CaMKIIa) promoter which has been reported to preferentially affect excitatory neurons.^57,58^ Further characterization of the transcriptional regulation of SUR1-TRPM4 expression in epilepsy, its effects on expression of other genes, and the specific contributions of expression in excitatory versus inhibitory neurons to the net excitability of the overall seizure network is needed.

The mechanism by which SUR1-TRPM4 channel activation occurs in epileptic neurons remains unclear. Under normal physiologic conditions, SUR1-TRPM4 channel open probability is minimal. However, channel open probability is elevated by increased intracellular Ca^2+^ and reduced ATP, both of which occur in epilepsy. The dynamic ranges for SUR1-TRPM4 channel activation for each of these metabolites are roughly 0.1–1 μM for Ca^2+^ and 0.1–3 μM for ATP.^53^ Our overexpression data suggest that SUR1-TRPM4 expression increases interictal excitability and potentiates ictal activity. In the chronic interictal state, hippocampal neurons from pilocarpine-induced SE models show elevated baseline Ca^2+^ levels (0.325 ± 0.04 μM 1 year after SE compared with 0.09 ± 0.02 μM in controls),^19,59^ corresponding to increasing channel open probability from ∼20% to ∼60% based on the open probability curves for SUR1-TRPM4 in response to Ca^2+^ concentration.^53^ During ictal events, Ca^2+^ levels rise further, and impaired clearance exacerbates hyperexcitability.^19,59^ Although intracellular ATP levels in chronic epileptic neurons are not well-characterized, epileptic brain tissues are known to be hypometabolic, with reduced ATP synthesis as reported in patient and rodent epileptic brains.^60,61^ Transient ATP decreases to around 1 mM have been documented during low Mg^2+^-induced hyperactivity, which corresponds to increasing SUR1-TRPM4 open probability from ∼20% to ∼50%.^53,62-65^ The interplay between elevated Ca^2+^ and reduced ATP likely creates favourable conditions for neuronal SUR1-TRPM4 activation, during hyperexcitability in epilepsy and future studies should explore this dynamic further to clarify the channel’s role in seizure pathology.

Some limitations should be noted. First, the electrographic sorting of human resection tissue relied on intracranial EEG, which may have introduced tissue injury and altered protein expression. However, as both control and epileptic samples were implanted with electrodes, this confounding variable is unlikely to account for any differences in expression seen between samples. Second, although the patient cohort included in the present study is diverse in terms of epilepsy aetiology, all patients included in this study had temporal lobe epilepsy with mesial temporal sclerosis (Table 1). This suggests that while our findings are generalizable across multiple causes of epilepsy, there may be some limitations to their generalizability across non-temporal lobe or non-lesional epilepsies. Future evaluation of SUR1-TRPM4 expression in non-temporal lobe epilepsies, which usually occur due to structural lesions caused by cortical malformation, tumours or trauma rather than network-level cytoarchitectural alterations that underly mesial temporal sclerosis, is warranted.^66^ Third, we intentionally did not perform intracranial EEG monitoring during *in vivo* SUR1-TRPM4 inhibition experiments in mice to avoid artificially inflating seizure susceptibility through electrode implants.^67^ Consequently, subclinical electrographic seizures may have gone undetected. Future studies should further characterize electrographic responses to SUR1-TRPM4 inhibition in the PTZ kindling model.

Pathological neuronal SUR1-TRPM4 expression represents a novel channelopathy in epilepsy, characterized for the first time in human tissue, that significantly contributes to seizure-related hyperexcitability. Recent advances in genetic sequencing have identified ion channel dysfunction as a key contributor to epilepsy, driven by mutation or dysregulation from acquired factors.^68,69^ Unlike constitutively expressed epilepsy-associated ion channels and receptors,^69^ SUR1-TRPM4 is minimally expressed in the healthy brain, making its upregulation a distinct biomarker of epileptic tissue and targetable mechanism. Non-invasive imaging techniques for other protein biomarkers, such as PET to localize amyloid-β, tau or GFAP expression in the brain, are an important area of active clinical and translational research.^70-73^ If molecular probes for SUR1-TRPM4 detection using PET or another imaging technique with improved spatial resolution were developed in the future, non-invasive detection of SUR1-TRPM4 as a specific marker of epileptic tissue (Fig. 1) could theoretically be integrated into a multimodal presurgical evaluation to provide adjunctive information on seizure foci location prior to iEEG implantation. Moreover, upregulation of certain genetic variants of SUR1 or of TRPM4 may amplify seizure susceptibility, as found for increased susceptibility in traumatic brain injury and intracerebral haemorrhage.^74-76^ Our work underscores the therapeutic potential of SUR1-TRPM4 inhibition for seizure management, independent of the underlying mechanism of epileptogenesis. Pharmacological inhibition using agents such as GLYB, which is FDA approved for diabetes management and in clinical trials for stroke, TBI and ICH,^13,14,77-81^ offers a promising path towards rapid translation through clinical trials and should be investigated both as a stand-alone therapy and as a synergistic adjuvant for seizure reduction with current ASMs. Our findings demonstrate that SUR1-TRPM4 overexpression drives neuronal hyperexcitability and seizure susceptibility, suggesting that inhibition may reduce seizure burden across diverse causes of epilepsy. Future studies should further explore the clinical application of SUR1-TRPM4 inhibitors for epilepsy and other epileptogenic injuries.

## Supplementary Material

awaf435_Supplementary_Data

## Data Availability

All data are available in the main text or the Supplementary material. All reported data will be shared by the corresponding author upon request. All original code has been deposited at Github and is publicly available at https://doi.org/10.5281/zenodo.14908460 as of the date of publication.
