# Supplementary material for: SUR1-TRPM4 is expressed in human epilepsy and promotes neuron hyperactivity and seizures in rodents
Source: Brain. 2025 Nov 15;149(6):2124–38. doi: 10.1093/brain/awaf435 (PMC13232044; doi:10.1093/brain/awaf435)
Supplement: awaf435_Supplementary_Data [file awaf435_supplementary_data.pdf]

Supplementary Methods:

Trpm4$^{fl/fl}$;+CaMKIIa-cre/ERT2 Mouse Generation and Induction

To generate conditional neuron-specific *Trpm4* KO mice (*Trpm4*$^{fl/fl}$;+CamkIIa-cre/ERT2), we bred CaMKIIa-cre/ERT2 mice bred on a C57Bl/6J background (The Jackson Laboratory: strain # 012362) with a custom-generated *Trpm4*$^{fl/fl}$ mouse line bred on the same background (Cyagen).[1] Cre-driven knock-out was targeted using Crispr/Cas9 guide RNAs to exons 10-12 of the mouse *Trpm4* gene (NCBI Reference Sequence: NM_175130), which results in a frameshift of the gene. WT littermate mice (*Trpm4*$^{fl/fl}$; -CaMKIIa-cre/ERT2) were used as controls. To induce *Trpm4* KO, we administered tamoxifen (75mg/kg in corn oil IP) once daily for 5 days, and experimentation was not initiated until 1-2 weeks after the final tamoxifen dose to allow sufficient time for Cre recombination to occur.

Quantitative Polymerase Chain Reaction (qPCR) for Gene Knock-out Validation

To validate effective constitutive and conditional *Trpm4* KO and constitutive *Kcnj11* KO *in vivo*, we employed qPCR with PCR primers targeted to the KO region of each respective gene of interest in each condition (Supplementary Table 1). For constitutive KO experiments, we extracted whole brains as described above, then extracted, purified, and amplified mRNA using standard procedures as previously described.[2] For neuron-specific *Trpm4* KO experiments, we first enriched for neurons after whole brain extraction using the Miltenyi Biotec adult mouse neuron isolation protocol (Miltenyi Biotec, Gaithersburg, MD, USA, Cat: 130-126-602), then extracted mRNAs from neuron enriched and depleted fractions and performed mRNA preamplification as

described by the manufacturer (ThermoFisher, Cat: 11752048). In all KO conditions, relative CT values were normalized to *Hprt* as the housekeeping gene.

Continuous video-electroencephalogram (vEEG)

To characterize our standard PTZ kindling model, we employed continuous vEEG monitoring to assess electrographic and behavioral seizure activity in mice. These animals were not used for protein expression or PTZ dose-escalation experiments due to the potential injury response induced by electrode implants. First, we implanted mice with intracranial electrodes system (Pinnacle Technology DE LLC, 8201) consisting of two subdural cortical contacts and reference/ground leads. Cortical contact 1 was positioned in the right primary motor cortex 1 mm anterior and 1 mm lateral to bregma, cortical contact 2 was positioned in the ipsilateral parietal cortex 1mm posterior and 1mm lateral to bregma, the reference lead was placed 1mm posterior and 1 mm lateral to bregma (contralateral to cortical contact 2), and the ground lead was placed 1 mm anterior and 1 mm lateral to bregma (contralateral to cortical contact 1). Starting 3 days after implantation, we recorded PTZ kindling vEEG responses for up to 30 minutes after PTZ administration, sampling a continuous voltage trace at 2000 Hz alongside a live video recording. We characterized electrographic seizures as high frequency, high amplitude continuous spike-wave discharging lasting at least 10 seconds.[3] We also monitored behavioral responses using the Racine Scale as above.[4]

Primary Culture Generation, Seeding, and Maintenance

Rat cortical cultures for *in vitro* TRPM4 inhibitor experiments were generated as we previously described.[5] In short, we anesthetized newborn rat pups with 5% isoflurane with 0.8 L/min O_2 flow rate, then were isolated, dissociated, and plated rat cortices onto Axion Cytoview

multi-electrode array (MEA) 96-well plates (Axion Biosystems, Atlanta, GA, M768-tMEA-96W) coated with Poly-D-Lysine (PDL) (1 mg/ml in borate buffer pH 8.4). We seeded cortical cultures at 200k cells/well in seeding media (5% FBS, 1x B-27 supplement, 2x antibiotic/antimycotic mix, 5 mM HEPES, and 1.2 mM L-glutamine in Neurobasal medium, pH 7.4). 24 hours after seeding to allow cell adherence, a full media change from seeding to maintenance media (5% FBS, 1x B-27 supplement, 1x antibiotic/antimycotic mix, 5 mM HEPES, and 1.2 mM L-glutamine in Neurobasal medium, pH 7.4) was performed on the cells, and they were maintained in maintenance medium with media changes every four days until the beginning of the treatment period on day *in vitro* (DIV) 17.

For SUR1-TRPM4 overexpression experiments, we isolated cortices from male and female E18 Sprague-Dawley rat embryos, dissociated, and plated on glass-bottom dishes (MatTek P35G-1.5-14-C) coated with poly-L-lysine in plating media (Neurobasal medium supplemented with 2% B27, 2 mM Glutamax, 50 U/mL Penicillin-Streptomycin, and 5% bovine serum) as previously described.[6] We seeded neurons at 120 k cells/well. At DIV 4, we treated cultures with 2 mM (+)-5-fluor-2'-deoxyuridine for 24 h, followed by exchange of growth media (Neurobasal medium supplemented with 2% B27, 2 mM Glutamax, 50 U/mL Penicillin-Streptomycin). On DIV7, we transfected neurons with a plasmid encoding SUR1 and TRPM4 proteins linked by a short amino acid sequence and co-expressed with a GFP reporter (pEF1α-SUR1-TRPM4-IRES-AcGFP1; WT).[7] Prior work from our lab demonstrated that this fusion protein expresses well and is fully functional after transfection.[7] As a control, the WT plasmid was also used to generate a plasmid encoding a non-functional SUR1-TRPM4 protein (pEF1α-SUR1-TRPM4D980AΔ-IRES-AcGFP1; Δ) by inducing a point mutation from Asp980 to Ala in the mouse *Trpm4* region of

SUR1-TRPM4, equivalent to the described human D984A mutation.[8] Ca^{2+} imaging experiments were performed on DIV9.

Supplementary Figures:

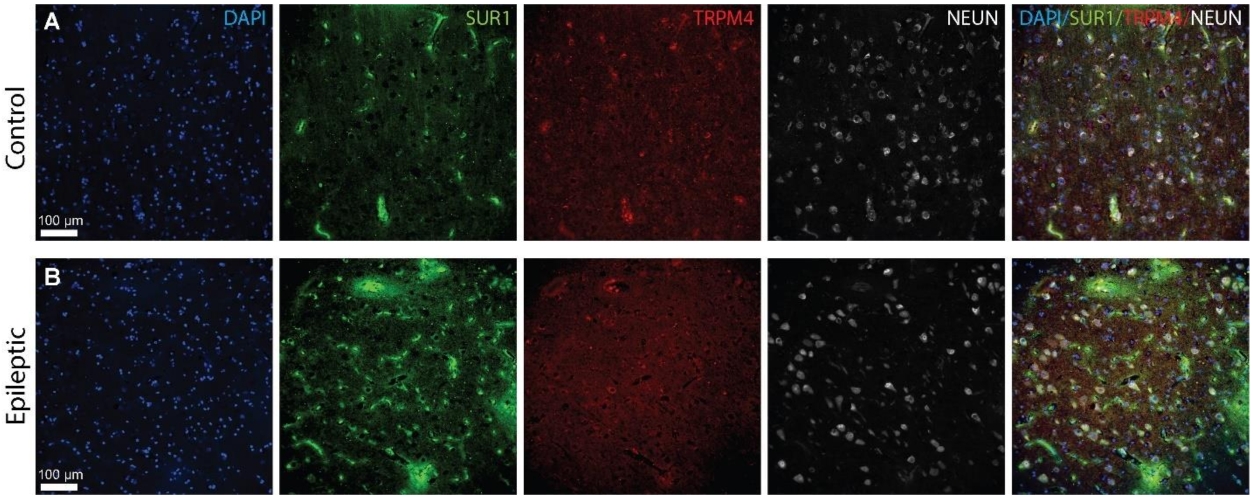

Supplementary Fig. 1: Representative 20x magnification images for SUR1-TRPM4 immunolabeling in human samples. For control (**A**) and epileptic (**B**) samples, single channel images DAPI (blue), SUR1 (green), TRPM4 (red), and NEUN (white), as well as combined channel images are present.

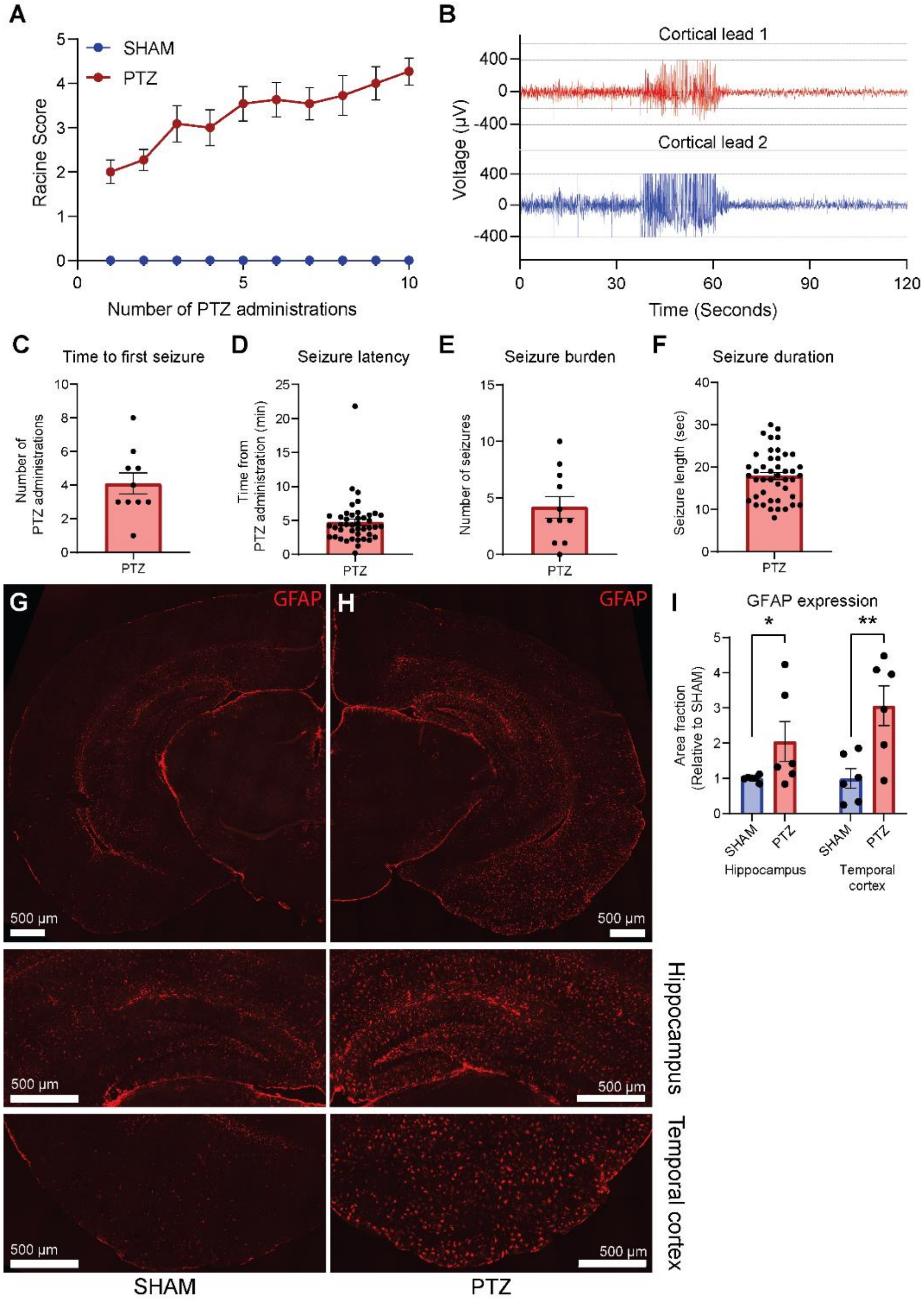

Supplementary Fig. 2: Characterization of the PTZ kindling model. (**A**) Seizure severity scores (Racine) demonstrate that mice undergoing PTZ kindling experience progressively worsening seizure phenotypes over the course of induction. SHAM mice do not experience seizures. (**B**) Representative seizure captured using intracranial cortical electrode EEG recording after PTZ induction. (**C-F**) Seizure characteristics of PTZ kindling, including the number of PTZ

administrations to first seizure (**C**), acute seizure latency after PTZ administration (**D**), number of seizures experienced per animal (**E**), and the duration of seizure events (**F**). (**G-H**) Immunofluorescent images of GFAP expression within SHAM (**G**) and PTZ (**H**) mice, displayed across the extent of the entire labeled sections, as well as locally within areas of high GFAP expression (hippocampus and temporal cortex). (**I**) Quantification of GFAP expression demonstrates significant upregulation within hippocampus (*p=0.047, t=1.846, df=10*) and temporal cortex *(p=0.004, t=3.28, df=10*) in PTZ mice compared to SHAMs. Data are represented as mean ± SEM. (*n=6* mice, **p<0.05*, ***p<0.01*, one-tailed unpaired t-test).

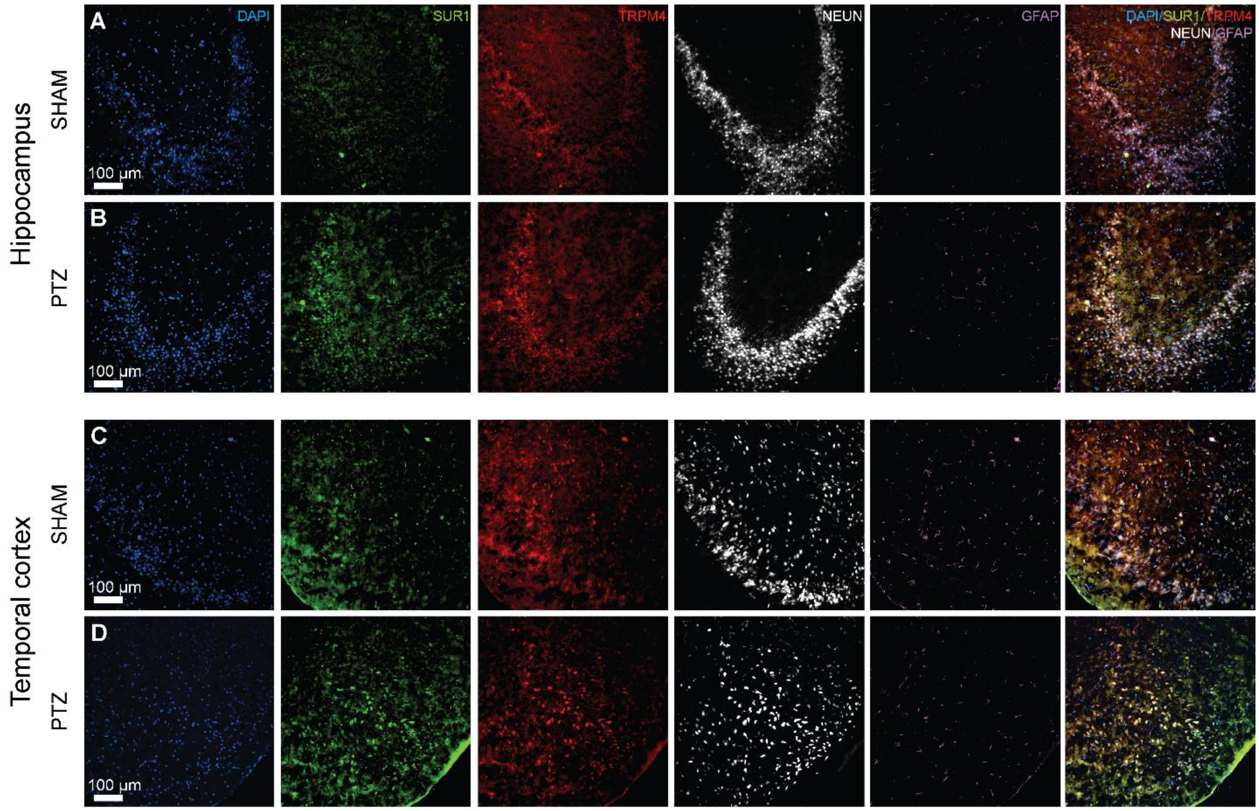

Supplementary Fig. 3: Representative 20x magnification images for SUR1-TRPM4 immunolabeling in PTZ kindled mouse brain. Hippocampal (**A, B**) and temporal cortical (**C, D**) 20x fields for SHAM (**A, C**) and PTZ (**B, D**) mouse brain sections are shown. Single channel

images for DAPI (blue), SUR1 (green), TRPM4 (red), NEUN (white), and GFAP (purple), as well as combined channel images are present.

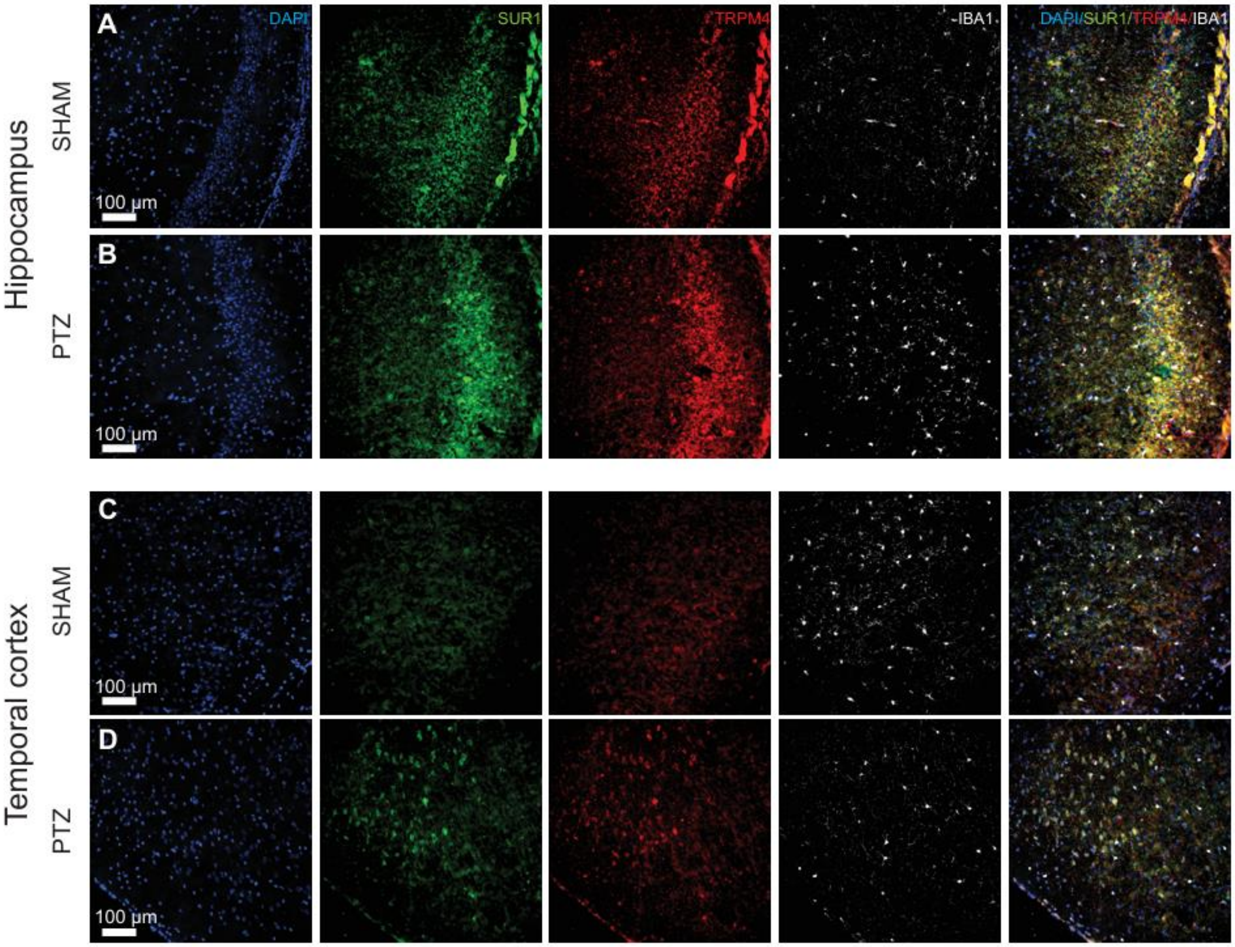

Supplementary Fig. 4: Representative 20x magnification images for SUR1-TRPM4 colocalization analysis with IBA1 in PTZ kindled mouse brain. Hippocampal (**A, B**) and temporal cortical (**C, D**) 20x fields for SHAM (**A, C**) and PTZ (**B, D**) mouse brain sections are shown. Single channel images for DAPI (blue), SUR1 (green), TRPM4 (red), IBA1 (white), as well as combined channel images are present.

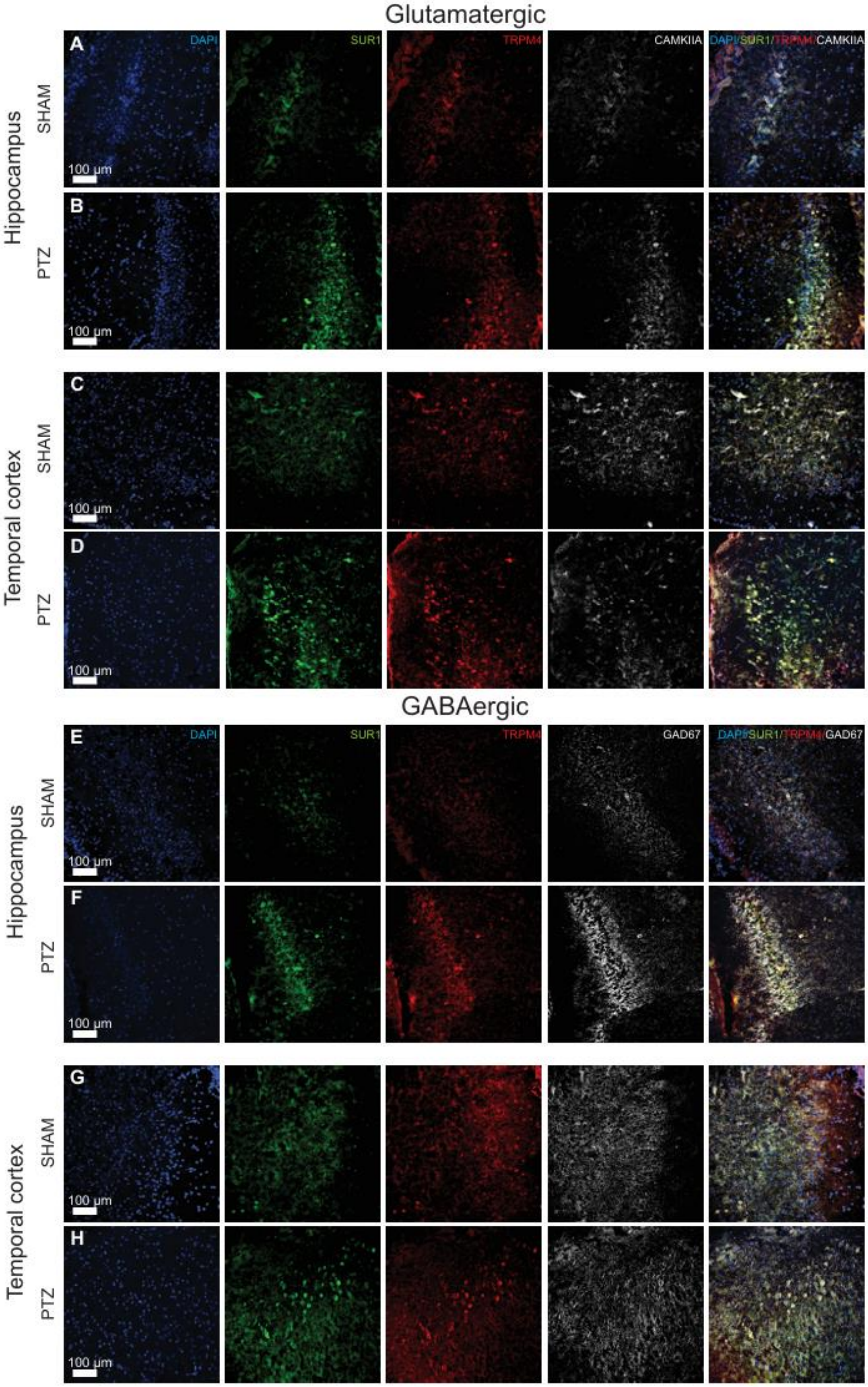
Glutamatergic
A
DAPI
SUR1
TRPM4
CAMKIIA
DAPI/SUR1/TRPM4/CAMKIIA
B
C
D
Hippocampus
Temporal cortex
SHAM
PTZ
100 µm
GABAergic
E
DAPI
SUR1
TRPM4
GAD67
DAPI/SUR1/TRPM4/GAD67
F
G
H

Supplementary Fig. 5: Representative 20x magnification images for SUR1-TRPM4 neuronal subtype analysis in PTZ kindled mouse brain. Hippocampal (**A, B, E, F**) and temporal cortical (**C, D, G, H**) 20x fields for SHAM (**A, C, E, G**) and PTZ (**B, D, F, H**) mouse brain sections are shown. Single channel images for DAPI (blue), SUR1 (green), TRPM4 (red), CAMKIIA or GAD67 (white), as well as combined channel images are present.

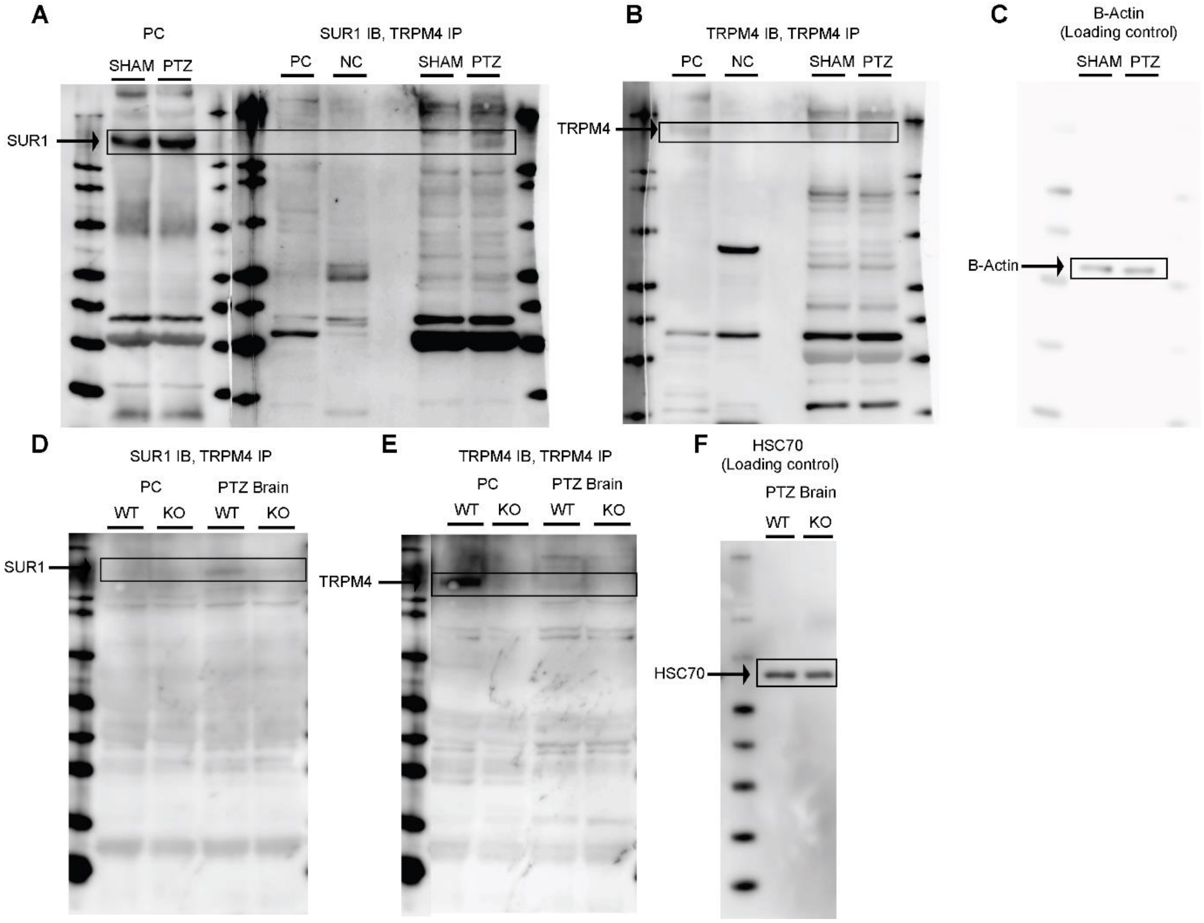

Supplementary Fig. 6: Representative western blot results for co-immunoprecipitation (co-IP) experiments. (**A-C**) Co-IP for SUR1 (**A**) is detected on immunoblot (IB) after TRPM4 immunoprecipitation (IP). This signal appears darker in the PTZ sample compared to SHAM and aligns with the positive control (PC), which in this case is SUR1 IP from mouse brain. (**B**) Successful TRPM4 IP is verified by TRPM4 IB. Note TRPM4 densitometry signals appear similar

between SHAM and PTZ samples, suggesting equal IP of TRPM4. TRPM4 signals align well with the PC signal (TRPM4 IP from mouse lung lysate) and no TRPM4 signal is seen in the negative control (NC) (IP from mouse lung lysate using a non-specific chicken IgG in place of chicken TRPM4 antibody). (**C**) Loading control for total brain lysates taken from SHAM and PTZ samples prior to IPs demonstrate equal protein amounts. (**D-F**) SUR1-TRPM4 co-IP experiment conducted as above in either constitutive *Trpm4* knock-out (KO) or wildtype (WT) littermate control mice samples after PTZ kindling. Note that SUR1 signal (**D**) is detected in PTZ brain samples of WT mice, in which TRPM4 IP is successful (**E**), but no SUR1 signal is detected after TRPM4 IP in *Trpm4* KO mice in which no TRPM4 is detected after IP. (**F**) Loading control validates equal protein amounts across PC (mouse lung) and PTZ brain samples.

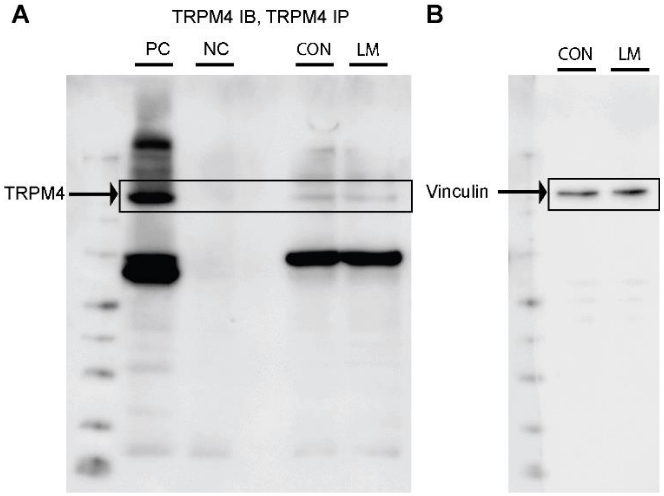

Supplementary Fig. 7: Western blot detection of TRPM4 in low Mg^{2+} in vitro model. (**A**) Representative western blot demonstrating expression of TRPM4 in rat mixed cortical culture one day after either control or low Mg^{2+} treatments. TRPM4 signal intensity appears similar between conditions and aligns with the positive control (PC), rat lung tissue. (**B**) Loading control signals (vinculin) appear similar between samples, demonstrating equal protein load.

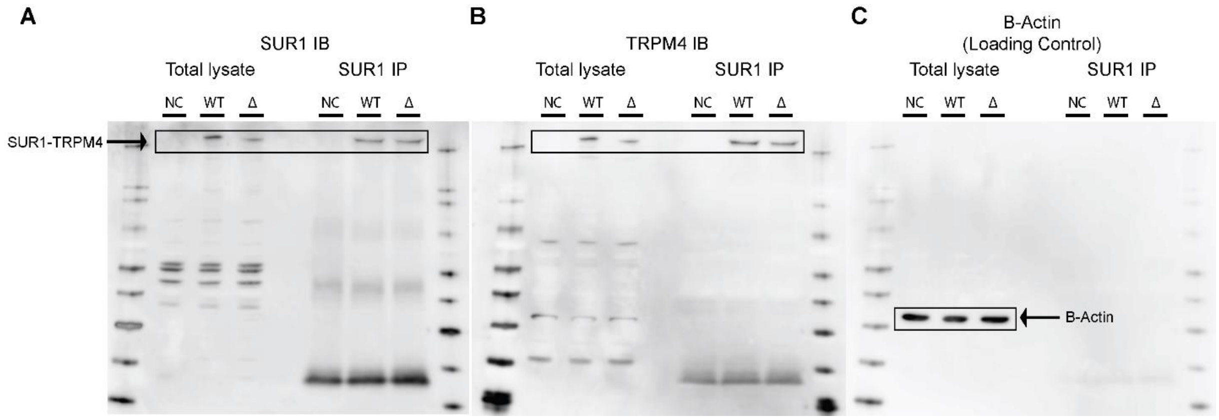

Supplementary Fig. 8: Western blot validation of SUR1-TRPM4 fusion protein overexpression. (**A**) Immunoblot (IB) for SUR1 using either total lysates or lysates after SUR1 immunoprecipitation (IP) from cos7 cells transfected with either wildtype (WT) SUR1-TRPM4 overexpression plasmid or non-functional (Δ) SUR1-TRPM4 plasmid, or non-transfected negative control (NC) cells. Bands are detected in the expected position for fused SUR1-TRPM4 protein in both the total lysate and SUR1 IP conditions for both WT and Δ transfected cells, but not for NC cells. (**B**) TRPM4 IB of the same blot as A after stripping demonstrates successful TRPM4 detection in the same positions as SUR1. (**C**) Loading control IB of the same blot demonstrates equal protein loading across all 3 cell conditions, and depletion of loading control signal in SUR1 IP conditions suggests SUR1 IP is specific.

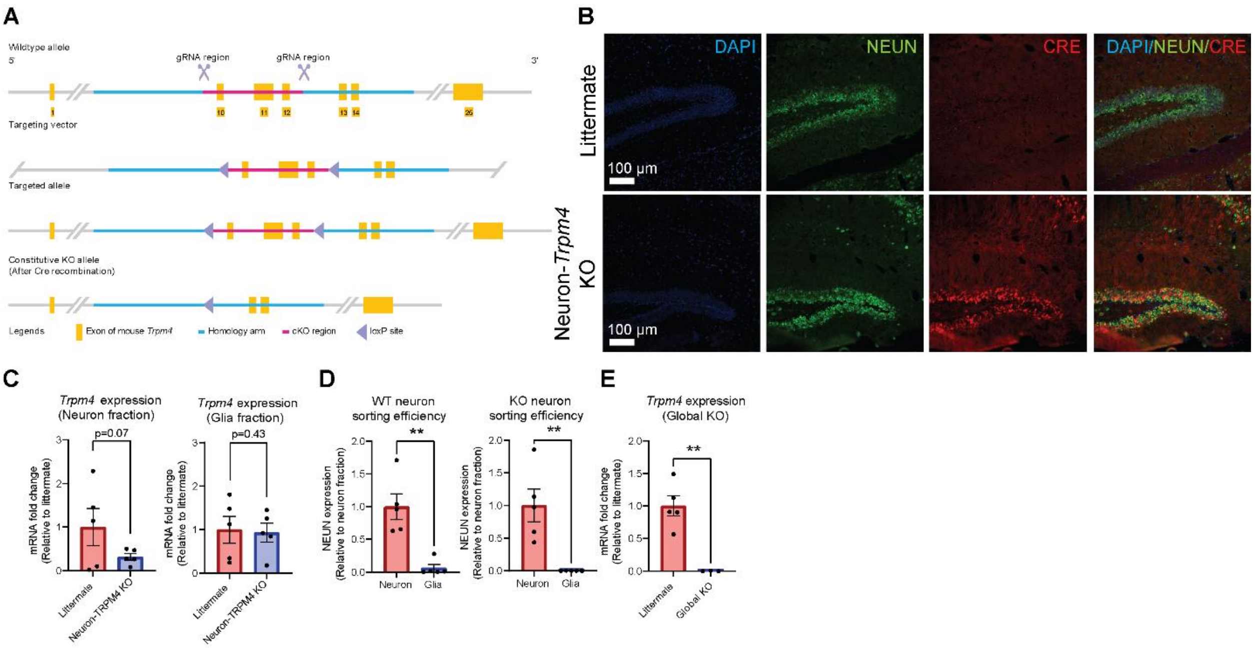

Supplementary Fig. 9: Validation of Trpm4 deletion in constitutive and conditional Trpm4 KO mice. (**A**) Schematic of targeted allele, and of the allele after CRE recombinase in conditional *Trpm4* KO mice. (**B**) Immunofluorescent labeling for CRE expression demonstrates successful induction of CRE (red) within NEUN+ neurons (green) of *Trpm4* KO mice, but not of WT littermate control mice. (**C**) Neuron-enriched and glial (neuron negative fraction) isolations from whole brains generated by magnetic sorting were analyzed by qPCR for *Trpm4* mRNA. Near-significant reduction of *Trpm4* was seen in neuron-*Trpm4* KO compared to littermates in neuron-enriched fractions (*p=0.07, t=1.57, df=8*), but not in glial fractions (*p=0.43, t=0.18, df=8*). (**D**) Efficiency of neuronal enrichment was assessed for both littermate (WT) and KO mouse brain separations using qPCR for *Rbfox3* (NEUN) mRNA. Significantly elevated NEUN expressions were seen in neuron fractions compared to glial fractions for both groups (WT: *p=0.002, t=4.63, df=8*; KO: *p=0.004, t=4.01, df=8*). (**E**) Whole brain qPCR for *Trpm4* mRNA demonstrates significant reduction (*p=0.001, t=4.92, df=6*) in *Trpm4* mRNA expression in constitutive KO mice compared to littermate controls. Data are represented as mean ± SEM. (Conditional KO: *n=5* mice,

Conditional WT: *n=5* mice; Constitutive KO: *n=3* mice, Constitutive WT: *n=5* mice; ***p<0.01*, one-tailed unpaired t-test).

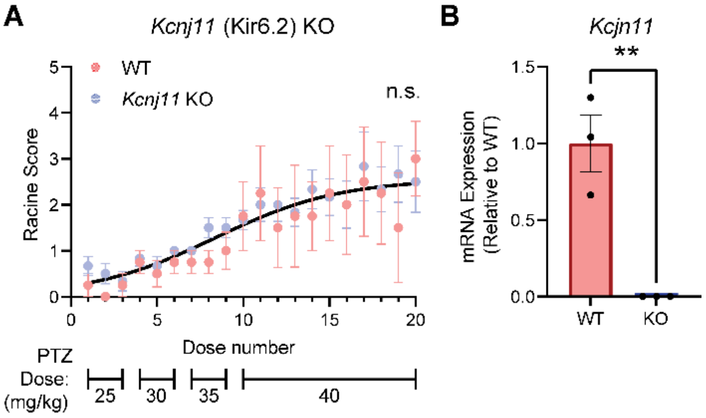

Supplementary Fig. 10: Kcnj11 (KIR6.2) KO has no effect on PTZ kindling. (**A**) Racine behavioral scoring demonstrates WT (red) (*n=4*) and KIR6.2 KO (*n=6*) mice exhibit similar responses to PTZ kindling (*p=0.52*, logistic growth non-linear regression analysis). (**B**) Assessment of whole brain *Kcnj11* mRNA expression by qPCR demonstrates successful depletion of *Kcnj11* in KO mice (*n=3* per group, *p=0.003, t=5.41, df=4*, one-tailed unpaired t-test). Data are represented as mean ± SEM. (n.s.: non-significant, **p<0.01).

References for Supplementary Materials:

1. L. Madisen, T. A. Zwingman, S. M. Sunkin, S. W. Oh, H. A. Zariwala, H. Gu, L. L. Ng, R. D. Palmiter, M. J. Hawrylycz, A. R. Jones, E. S. Lein, H. Zeng, A robust and high-throughput Cre reporting and characterization system for the whole mouse brain. *Nat Neurosci* **13**, 133-140 (2010).
2. D. B. Kurland, V. Gerzanich, J. K. Karimy, S. K. Woo, R. Vennekens, M. Freichel, B. Nilius, J. Bryan, J. M. Simard, The Sur1-Trpm4 channel regulates NOS2 transcription in TLR4-activated microglia. *J Neuroinflammation* **13**, 130 (2016).

3. Q. Wang, N. de Prisco, J. Tang, V. A. Gennarino, Protocol for recording epileptiform discharges of EEG and behavioral seizures in freely moving mice. *STAR Protoc* **3**, 101245 (2022).

4. R. J. Racine, Modification of seizure activity by electrical stimulation. II. Motor seizure. *Electroencephalogr Clin Neurophysiol* **32**, 281-294 (1972).

5. A. Ksendzovsky, M. Bachani, M. Altshuler, S. Walbridge, A. Mortazavi, M. Moyer, C. Chen, I. Fayed, J. Steiner, N. Edwards, S. K. Inati, J. Jahanipour, D. Maric, J. D. Heiss, J. Kapur, K. A. Zaghloul, Chronic neuronal activation leads to elevated lactate dehydrogenase A through the AMP-activated protein kinase/hypoxia-inducible factor-1α hypoxia pathway. *Brain Commun* **5**, fcac298 (2023).

6. C. A. Dean, S. R. Metzbower, S. K. Dessain, T. A. Blanpied, D. R. Benavides, Regulation of NMDA Receptor Signaling at Single Synapses by Human Anti-NMDA Receptor Antibodies. *Front Mol Neurosci* **15**, 940005 (2022).

7. S. K. Woo, M. S. Kwon, A. Ivanov, V. Gerzanich, J. M. Simard, The sulfonylurea receptor 1 (Sur1)-transient receptor potential melastatin 4 (Trpm4) channel. *J Biol Chem* **288**, 3655-3667 (2013).

8. B. Nilius, J. Prenen, A. Janssens, G. Owsianik, C. Wang, M. X. Zhu, T. Voets, The selectivity filter of the cation channel TRPM4. *J Biol Chem* **280**, 22899-22906 (2005).

Supplementary Tables:

Supplementary Table 1: Primer sequences used for qPCR genotype validation experiments.

Gene (All Mouse)	Forward (5'-3')	Reverse (5'-3')	Amplicon Length	Exon-Exon Junction
Trpm4 (Global KO)	CCTCTCATCTACACCAACCTTATC	CATTGATGCTGCTGTCCATATC	94	15-16
Trpm4 (Neuron KO)	ATCGTTTTGAGGGCTCTTGTG	TTGGATGTCCCCACGGAAAA	132	9-10
Kcnj11	TTCACCATGTCCTTCCTGTG	AAGGAGTGGATGCTTGTGAC	128	N/A (Gene only has 1 exon)
Rbfox3	GTTGCCTACCGGGGTGCACAC	TGCTCCAGTGCCGCTCCATAAG	110	11-12
Hprt	CTGGTGAAAAGGACCTCTCGAAG	CCAGTTTCACTAATGACACAAACG	146	6-7
